# Inhibition of rhizobial cheaters by the host *Medicago truncatula* involves repression of symbiotic functions and induction of defense

**DOI:** 10.1111/nph.70494

**Published:** 2025-09-05

**Authors:** Min Chen, Axelle Raisin, Natalie Judkins, Pierre‐Marie Allard, Emmanuel Défossez, Michael Stumpe, Inmaculada Yruela, Manuel Becana, Didier Reinhardt

**Affiliations:** ^1^ Department of Biology University of Fribourg Chemin du Musée 10 1700 Fribourg Switzerland; ^2^ Department of Plant Biology Estación Experimental de Aula Dei, CSIC Avenida Montañana 1005 50059 Zaragoza Spain

**Keywords:** legume–rhizobium symbiosis, *Medicago truncatula*, *nifH*, nitrogen fixation, nodulation, sanctioning, *Sinorhizobium meliloti*

## Abstract

- In symbiotic plant–microbe interactions, the host invests considerable amounts of resources in the microbial partner. If the microbe does not reciprocate with a comparable symbiotic benefit, it is regarded as a cheater. The host responds to cheaters with negative feedback mechanisms (sanctions) to prevent fitness deficits resulting from being exploited. We study sanctioning in the symbiosis between *Medicago truncatula* and the nitrogen‐fixing rhizobium *Sinorhizobium meliloti*.
- We manipulated the exchange of resources between the partners in three ways: by using mutant rhizobia defective in nitrogenase; replacing nitrogen in the atmosphere with argon gas; and supplying rich nitrogen fertilizer to the host. We follow the consequences of simulated cheating by examining the metabolome and proteome of both partners.
- We find that sanctioning occurs at multiple levels. In particular, we observe repression of essential symbiotic functions and changes in central metabolism that are likely to be relevant for microbial fitness and that could therefore contribute to sanctioning. In addition, sanctioning triggers a broad panel of defense markers.
- A thorough understanding of the multilevel phenomenon of sanctioning will be essential for its genetic dissection and for the breeding of elite legume crops with efficient symbiosis.

## Introduction

Endosymbioses with microbial partners that confer a metabolic advantage are extraordinarily successful in plants, with the founding event being the domestication of photosynthetic cyanobacteria that became permanent cellular constituents, the chloroplasts (plastids in nonphotosynthetic tissues) (Zimorski *et al*., 2014). In addition, the majority of plants engage in an endosymbiotic relationship with arbuscular mycorrhizal fungi that provide their host with various essential mineral nutrients (Smith & Read, 2008). Some lineages of the angiosperms (Fabales, Fagales, Cucurbitales, and Rosales) have evolved an additional symbiosis with nitrogen‐fixing (N‐fixing) soil bacteria (rhizobia), in which the bacteria supply the host with ammonia in exchange for fixed carbon. A well‐known example of this kind is the legume–rhizobium symbiosis (LRS) (Oldroyd *et al*., 2011).

While LRS provides critical advantages to both partners, the dynamics of the interaction are very asymmetrical: Plants have usually one generation per year, while bacteria can potentially divide every few hours. Infection of the root with single rhizobial cells gives rise to nodules that harbor many millions of bacterial descendants within a few weeks (Quides *et al*., 2021). This raises the possibility that spontaneous mutations in N‐fixation genes may generate selfish rhizobia that would enjoy a selective advantage due to the relief from the metabolic burden associated with the energetically costly N‐fixation process (West *et al*., 2002). The ensuing risk for the host is twofold: The individual plant may suffer from a disadvantage compared with conspecific individuals of the same population that harbor better symbionts; and the local plant population as a whole may increasingly suffer from a progressive decline in N‐fixation capacity in the rhizobial population. Hence, it has been predicted that, for the host species, detection of cheaters and their inhibition (or the specific promotion of good mutualists) is of primordial importance for long‐term persistence of the symbiotic interaction and for survival of the species (Kiers *et al*., 2008). Indeed, several studies have shown that legume hosts can distinguish rhizobia according to their symbiotic performance and selectively sanction cheaters, thereby enforcing bacterial mutualism (West *et al*., 2002; Kiers *et al*., 2003; Kiers & van der Heijden, 2006; Oono *et al*., 2009; Daubech *et al*., 2017; Westhoek *et al*., 2017; Berrabah *et al*., 2018). However, the underlying mechanisms remain poorly understood (Kiers *et al*., 2008; Oono *et al*., 2009; Sachs *et al*., 2018).

Sanctioning requires that the host plant can recognize cheaters and that it can exert a negative feedback onto them (Denison, 2000; Simms & Taylor, 2002; West *et al*., 2002; Kiers & van der Heijden, 2006; Kiers *et al*., 2008; Kiers & Denison, 2008). Hence, the following questions arise: (1) What are the metabolic consequences of cheating for the host, and which molecule(s) may serve as signals for cheating? (2) Which mechanisms are activated in the host plant to sanction cheaters? and (3) At which level does sanctioning act (whole root system, individual nodules, or individual infected host cells)? The first answer is likely to involve intermediates or end products of N assimilation, such as ammonia or Gln (Denison, 2000; Schulte *et al*., 2021). The second answer can potentially involve direct inhibition of bacterial growth and cell division, reduced allocation of C (or other essential nutritional requirements), or induction of a defense response involving antibacterial proteins and metabolites. The answer to the third question may be that sanctioning occurs at the individual nodule level (Westhoek *et al*., 2021; Underwood *et al*., 2024), although it has been reported that in nodules inhabited by more than one rhizobial clone, the respective regions of the nodule can be sanctioned according to the rhizobial service; hence, invoking mechanisms of recognition and action act in a cell‐autonomous fashion (Checcucci *et al*., 2016; Regus *et al*., 2017). Interestingly, rhizobia of intermediate N‐fixation efficiency can be sanctioned or not, depending on whether they share a host plant with a better or worse competing rhizobium, respectively, indicating that sanctioning is conditional (Westhoek *et al*., 2021; Underwood *et al*., 2024). Taken together, these results show that legumes have evolved tightly regulated mechanisms to assess symbiotic service from their rhizobial partners and react with controlled rewards or sanctions to promote the growth of the best available partner.

The means by which sanctioning is implemented could involve the withdrawal of essential resources and/or the activation of antibacterial mechanisms. For both scenarios, evidence has been gathered: On the one side, sanctioning is associated with reduced supply of oxygen (Denison, 1998; Kiers *et al*., 2003) and carbon (Westhoek *et al*., 2021) to nodules, thereby curbing bacteroid activity and proliferation. On the other side, sanctioning involves activation of defense mechanisms against the rhizobial partner (Suganuma *et al*., 2003; Agtuca *et al*., 2020; Berrabah *et al*., 2024), which are normally repressed during nodule development (Berrabah *et al*., 2018).

To gain deeper insight into the sanctioning mechanisms of *Medicago truncatula*, we used a combined metabolomic and proteomic approach to characterize the interaction with its rhizobial partner under conditions of manipulated N‐fixation. We inoculated *M. truncatula* with either wild‐type (wt) *Sinorhizobium meliloti* (N‐fixing) or with *S. meliloti nifH* mutants (nonfixing) that lack an essential component of nitrogenase due to a transposon insertion in the *NifH* gene. In addition, we set up conditions under which plants were inoculated with wt rhizobia and subsequently cultured in N_2_‐free argon atmosphere (nonfixing). In a fourth treatment, rhizobial inoculation was followed by fertilization with high N levels that are known to inhibit nodulation (Oldroyd & Leyser, 2020). All three manipulations (*nifH*, argon, and high N) resulted in altered nodulation, indicative of sanctioning. Histological analyses revealed dramatic changes in nodule anatomy, bacterial occupancy, and carbon allocation in sanctioned nodules.

Nontargeted metabolomic analysis revealed characteristic metabolic shifts associated with manipulated N‐fixation, suggesting that sanctioning is associated with altered C and N metabolism in nodules. This notion was complemented by proteomic analysis, which revealed that sanctioning was associated with a general decrease in symbiotic markers such as leghemoglobins (Lbs) and nodule cysteine‐rich (NCR) peptides, and with activation of various defense markers. Finally, conspicuous changes in phosphorylation of Lb, of the symbiotic remorin SymRem1, and of the immune regulator RPM1‐interacting protein 4 (RIN4) suggest that sanctioning involves posttranslational modification of central regulators of symbiosis and defense. Proteomic evidence for sanctioning in the host is paralleled by changes in the rhizobial (phospho)proteome, which reflect the activation of stress‐coping mechanisms and the repression of symbiotic pathways in favor of markers associated with a free‐living lifestyle.

## Materials and Methods

### Cultivation of rhizobia and plant inoculation

*Sinorhizobium meliloti* strain 2011 was cultured in tryptone/yeast extract (TY) medium (5 g tryptone, 3 g yeast extract, and 0.4 g CaCl_2_ in 1 l of distilled water) in the presence of streptomycin (50 μg ml^−1^), and incubated for 48 h. For *S. meliloti 2011 nifH*::*Tn5*, neomycin (50 μg ml^−1^) was additionally included. Rhizobia were harvested by centrifugation at 1900 ***g*** for 15 min at 4°C and resuspended in 10 mM MgSO_4_. For plant inoculation, bacterial suspensions were adjusted to an OD_600_ of 0.2 with 10 mM MgSO_4_. Each plant was inoculated with 2 ml of bacterial suspension.

### Plant growth and sanctioning treatments for Dataset 1

Seeds of *Medicago truncatula* (Gaertn.) A17 were scarified by concentrated sulfuric acid for 10 min. After washing with sterile water three times, seeds were sterilized with concentrated Clorox (Reactolab SA) for 2 min, adding an equal volume of water for 1 min and rinsed with sterile water five times. Seeds submerged in sterile water were put in a gentle shaker for 4 h at room temperature (RT). Thereafter, seeds were spread on 0.8% plant agar plates for 3 d. Five seedlings each were transferred to sterilized box units consisting of two sterilized cylindrical plastic boxes linked by a cotton wick, with perlite in the upper jar and B&D nutrient solution with 0.5 mM KNO_3_ in the lower jar (Broughton & Dilworth, 1971). After two weeks, the plants were inoculated with wt *S. meliloti* 2011 or with *S. meliloti* 2011 *nifH*::Tn5 mutants. An additional treatment involved inoculation with wt rhizobia and culturing under a N_2_‐free argon mixture (80% argon, 20% O_2_, and 400 ppm CO_2_) purchased from Carbagas (https://www.carbagas.ch/de). All treatments involved four replicate pots with five plants each from which the nodules were pooled. Data clustering revealed a strong outlier among the *nifH* replicates; therefore, one of the four was excluded from further analysis.

### Plant growth and sanctioning treatments for Dataset 2 and metabolomics analysis

For metabolomics and tandem mass tag (TMT)‐labeled proteomics (Dataset 2), plants were inoculated and cultured as described previously under control conditions for 2 wk, followed by treatments for 1 wk under argon atmosphere, or high N fertilization (10 mM NH_4_NO_3_). Hence, following the inoculation, all plants were first grown in open air for 14 d and then transferred to sealed boxes. Six jars inoculated with the wt rhizobial strain were treated with argon atmosphere, while the remaining plants were treated with air. After 7 d of treatment, 100 mg nodules was harvested and pooled for each replicate pot. All treatments involved four replicate pots with five plants each from which the nodules were pooled.

For metabolomics analysis, nodules (100 mg) were ground to a fine powder under liquid nitrogen. To this powder, 1 ml of 80% acetonitrile was added and the nodule powder solution was sonicated twice, with each round lasting for 1 min. After letting the solution sit for 5 min on ice, the samples were centrifuged at 13 500 ***g*** for 3 min. The resulting supernatant (max 800 μl) was then collected for liquid chromatography and mass spectrometry analysis with two technical replicates. Pure standard solutions of the compounds of interest were injected for reference to verify predicted molecular identity by comparison of retention times and mass spectra. Statistical analysis involved one‐way analysis of variance (ANOVA) followed by Tukey's test (*P* < 0.05).

### Metabolomics analysis and data treatment

Chromatographic separation was performed on a Vanquish Flex UPLC system (Thermo Fisher Scientific, Waltham, MA, USA) interfaced with a Q‐Exactive Plus mass spectrometer (Thermo Fisher Scientific), using a heated electrospray ionization (HESI‐II) source. The Thermo Fisher Scientific Xcalibur 3.1 software was used for instrument control. The MS data were converted from.RAW (Thermo Fisher Scientific) standard data format to .mzML format using the MSConvert software, part of the proteowizard package (Chambers *et al*., 2012). The converted files were treated using MZmine v.4.1 (Pluskal *et al*., 2010). To analyze the spectral diversity of the profile collection, a molecular network was created on the GNPS website (http://gnps.ucsd.edu) using the .mgf spectra file generated at the previous step (Wang *et al*., 2016).

In addition to searching experimental spectral libraries, spectral matching against theoretical spectral libraries of natural products was employed to cover a wider spectral space (Allard *et al*., 2016). Furthermore, the taxonomical distance between the biological sources of candidate structures and the biological source of the annotated extracts was taken into account (Rutz *et al*., 2019). Thus, in addition to the spectral search performed at the molecular networking step against publicly available spectral libraries (see ‘Plant growth and sanctioning treatments for Dataset 2 and metabolomics analysis’ in the Materials and Methods section), a taxonomically informed metabolite annotation was performed (see Supporting Information, for more detail). Scripts for multivariate unsupervised and supervised analysis are available at the following GitHub repository: https://github.com/mapp‐metabolomics‐unit/biostat_toolbox. See Supporting Information for further detail.

### Proteomics analysis

Protein extraction was performed according to Marx *et al*. (2016) with minor modifications (Supporting Information). After protein extraction, equal amounts of proteins per sample were digested with LysC protease (ratio 50 : 1, for 3 h, at RT, final urea concentration 4 M) and trypsin (ratio 50 : 1, overnight, at RT, final urea concentration < 1 M). The next day, the samples were acidified using 50% trifluoroacetic acid (TFA) (final concentration app. 0.5%, pH < 2) and centrifuged at 1500 ***g*** for 10 min to remove precipitations. Peptides were purified by solid‐phase extraction (SPE) using HR‐X columns in combination with C18 cartridges (Macherey Nagel; https://www.mn‐net.com): wash buffer, 0.1% formic acid in deionized water; elution buffer, 80% acetonitrile and 0.1% formic acid in deionized water. Elutes were frozen in liquid nitrogen and lyophilized overnight. Purified peptides were fractionated by HpH reversed‐phase chromatography (Batth *et al*., 2014). LC‐MS/MS measurements were taken on a QExactive HFX mass spectrometer coupled to an EasyLC 1000 nanoflow‐HPLC (all Thermo Scientific). Peptides were separated on a fused silica HPLC‐column tip (I.D. 75 μm, New Objective, self‐packed with ReproSil‐Pur 120 C18‐AQ, 1.9 μm (Dr. Maisch; https://dr‐maisch.com) to a length of 20 cm) using a gradient of A (0.1% formic acid in water) and B (0.1% formic acid in 80% acetonitrile in water): Samples were loaded with 0% B with a flow rate of 600 nl min^−1^; peptides were separated by 5%–30% B within 85 min with a flow rate of 250 nl min^−1^. Spray voltage was set to 2.3 kV and the ion‐transfer tube temperature to 250°C (see Supporting Information, for further detail).

### Protein 3D‐structure modeling

3D homology modeling and structural alignment was driven with AlphaFold (Google DeepMind) and HHPred (https://toolkit.tuebingen.mpg.de/tools/hhpred) and Modeler using the structure of the *Glycine max* Lb*a* (PDB 1BIN) as a template. 3D alignment of the structural model of *M. truncatula* Lb2 (UniProt G7K1Z9) and the molecular crystal X‐ray structure was performed with PyMol 1.4.1 (Schrodinger LLC, https://www.pymol.org).

### Staining procedures and microscopy

Life‐and‐dead staining was performed as described (Nicoud *et al*., 2021). Briefly, fresh nodules were embedded in 6% agar and 0.2 M sucrose and sectioned with a vibratome HM 650S (Thermo Fisher Scientific). Sections were incubated with 5 μM Syto9 and 10 μM propidium iodide (PI) for 20–30 min and analyzed using a Leica SPE‐II confocal microscope. For basic fuchsin staining, nodules were fixed in 4% paraformaldehyde in 1× phosphate‐buffered saline (PBS) overnight. After three washes with 1× PBS, nodules were embedded in 6% agar and sectioned using the vibratome HM 650S. Sections were stained with 0.2% basic fuchsin in ClearSee (Kurihara *et al*., 2015) and imaged on a Leica SPE‐II laser scanning confocal microscope.

### Transmission electron microscopy (TEM)

Nodule samples were fixed by high‐pressure freezing and cryosubstitution followed by embedding in Spurr's resin. For transmission electron microscopic (TEM) analysis, ultrathin sections (70 nm) were prepared on a Reichert–Jung Ultracut E. Contrasting was performed with 2% (w/v) uranyl acetate (UO_2_(CH_3_COO)_2_) and lead citrate solution prepared according to Reynolds (1963). Images were acquired on a Philips Biotwin CM100 equipped with a digital camera (Morada Soft Imaging System) (see Supporting Information, for more detail).

Additional Materials and Methods are available in the online Supporting Information (Methods S1).

## Results

### Effects of sanctioning on nodule development

**Fig. 1:**
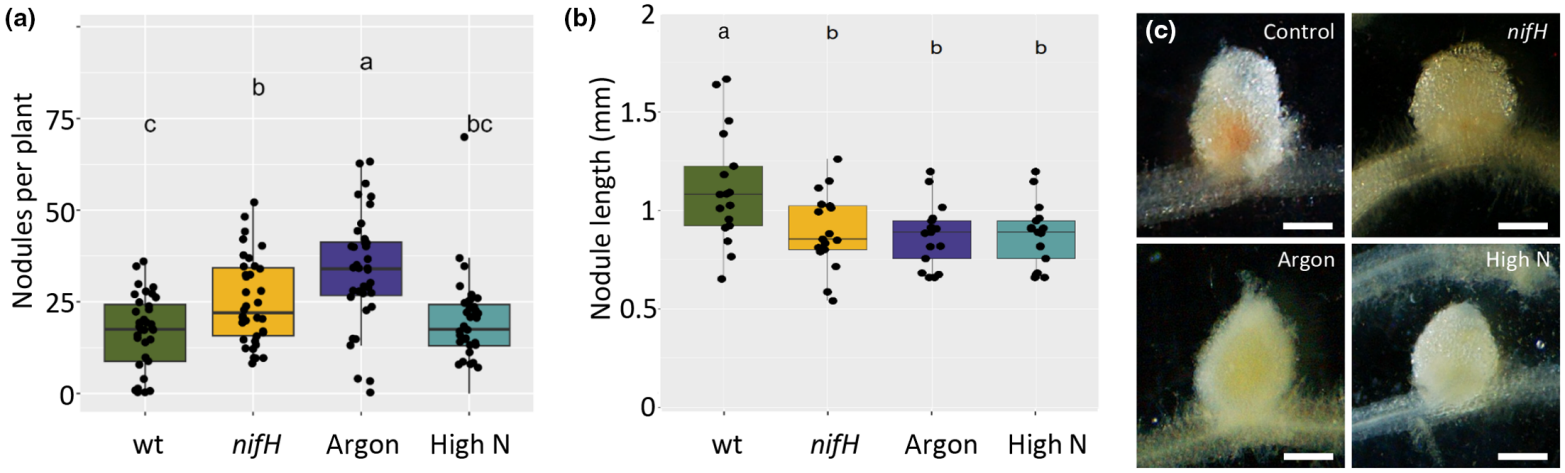
Sanctioning of *Medicago truncatula* against nodulation by *Sinorhizobium meliloti*. (a) Nodule number on *M*. *truncatula* root systems inoculated with wild‐type (wt) *S*. *meliloti*, with *nifH* mutants, and with wt bacteria under argon atmosphere or high‐N fertilization, as indicated. (b) Average length of nodules from plants as in (a). (c) Appearance of nodules as in (a, b) after 21 d of culture with rhizobia. Boxplots represent the median and the interquartile range; whiskers indicate the data range excluding outliers. Significance of differences was tested with one‐way ANOVA followed by Tukey's *post hoc* test (*n* = 36 for nodule number; *n* = 20 for nodule length). Letters above box plots indicate statistically significant differences between treatments. Bars, 0.5 mm.

To characterize the phenomenon of sanctioning under our experimental conditions, the macroscopic nodule phenotype was assessed in plants inoculated with the *nifH* mutant (Ruvkun *et al*., 1982), in plants grown under N‐free argon atmosphere, and in plants fertilized with high N levels. Both nonfixing conditions (*nifH*, argon) resulted in increased nodule numbers (Fig. 1a), indicating that autoregulation of nodulation is attenuated under nonfixing conditions. Nodule length was slightly reduced under sanctioning conditions (Fig. 1b); however, the most distinguishing feature of sanctioning in all experimental conditions was the strong reduction in nodule pigmentation compared with controls (Fig. 1c), indicating reduced Lb levels. The roundish shape of sanctioned nodules remained constant, while wt nodules elongated as expected for indeterminate nodules (Fig. S1).

### Bacteroid differentiation and survival in sanctioned nodules

Sanctioning generally results in reduced nodule size and fewer infected cells per nodule. In order to investigate the consequences of sanctioning for the endosymbiont, we employed an established staining protocol that assesses bacterial membrane integrity as a proxy for cellular viability; hence, it is known as live/dead staining (Haag *et al*., 2011; Guefrachi *et al*., 2014; Horvath *et al*., 2015; Kim *et al*., 2015; Barrière *et al*., 2017; Nicoud *et al*., 2021; Berrabah *et al*., 2023; Magne *et al*., 2024). Using the interaction with *nifH* mutants as the paradigmal example of sanctioning, we sectioned and stained nodules with the membrane‐permeable dye SYTO9 in combination with PI, which does not permeate intact plasma membranes (Mergaert *et al*., 2006).

**Fig. 2:**
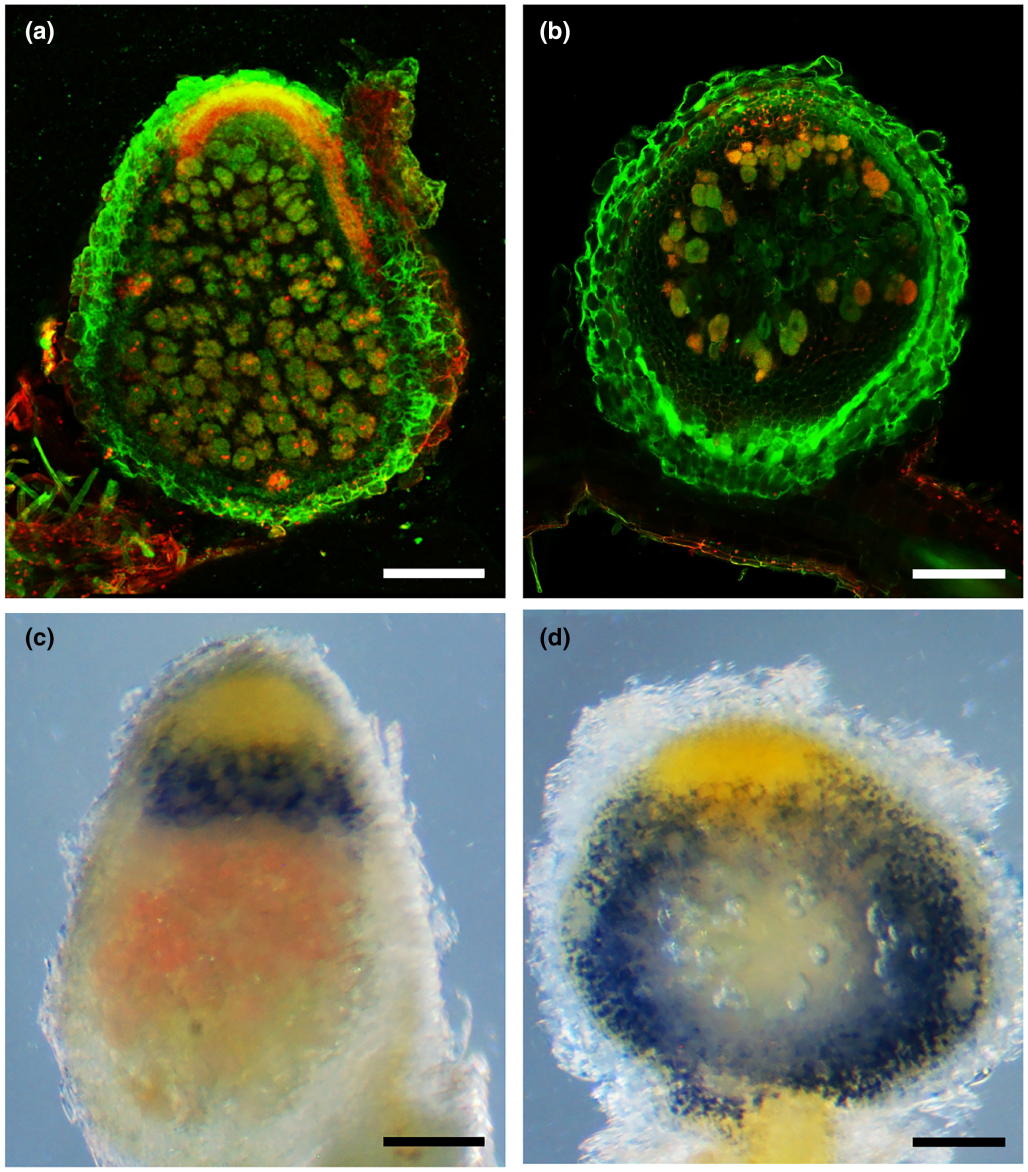
Bacteroid survival and starch accumulation in sanctioned nodules of *Medicago truncatula*. (a, b) Overview of nodules inhabited by wild‐type (wt) *Sinorhizobium meliloti* (a) and *nifH* mutant (b), respectively, 21 d after inoculation. Live/dead staining with SYTO9 (green) and PI (red) revealed intermediate membrane permeabilization in fully differentiated bacteroids under control conditions (a). In sanctioned nodules colonized by *nifH*, fewer colonized cells were detected, of which some showed stronger staining than in the wt (b). (c, d) Lugol staining for starch revealed a clearly defined purple band between the meristematic zone (yellow) and zone ZIII (pink) in wt nodules (c), whereas *nifH* nodules exhibited strong starch staining throughout large parts of the nodule proximal to the meristematic zone (d). Bars, 250 μm.

In wt nodules, most bacteroids were stained green by SYTO9 all along the developmental gradient (zones I, IId, IIp, IZ, III, and IV) (Figs 2a, S2a). In addition, the bacteroids exhibited a reddish hue that increased from the IZ to the senescence zone IV (Figs 2a, S2a,d), conceivably reflecting the increasing permeability of bacteroid membranes associated with terminal differentiation (Mergaert *et al*., 2006). Boiled nodules as a positive control for dead bacteroids showed much stronger red signal than any of the wt bacteroids (Fig. S2b), suggesting that membranes of the latter are only partially permeable. Staining in *nifH* nodules was less regular and showed a combination of cells with green and red staining as in the control nodules, and individual cells with stronger red staining, presumably representing senescent bacteroids (Figs 2b, S2c,e). The most obvious difference between *nifH* nodules and controls was the lower numbers of infected host cells, and a compromised zonation with a drastically reduced ZIII (Figs 2a,b, S2a,c), similar to nodule phenotypes observed in fix^−^ mutants such as *dnf2*, *dnf4*, *dnf7*, and *symCRK* (Berrabah *et al*., 2014; Horvath *et al*., 2015; Kim *et al*., 2015).

TEM analysis of wt and *nifH* nodules revealed that cells infected with wt rhizobia exhibited a well‐organized distribution of bacteroids around the central vacuole (Fig. S3a), while cells inhabited by *nifH* appeared rather disorganized and often lacked a central vacuole (Figs S1, S3b). In addition, infected (and noninfected) cells of *nifH* nodules had large starch granules along the cell periphery (Fig. S3b). Starch normally accumulates in cells of zone IIp to IZ and disappears in zone III, conceivably because of progressive degradation to fuel N‐fixation (Hostak *et al*., 1987; Vasse *et al*., 1990; Forrest *et al*., 1991). In nonfixing nodules, however, starch accumulates to higher levels (Hirsch *et al*., 1983; Hirsch & Smith, 1987; Forrest *et al*., 1991). We performed iodine staining to compare global starch accumulation patterns in wt and *nifH* nodules. Wild‐type nodules exhibited a distinct band of starch between the meristematic region and the fixation zone (ZIII), as described previously (Vasse *et al*., 1990), whereas the pink fixation zone itself appeared to be essentially starch‐free (Figs 2c, S4). In *nifH* nodules, starch was detected in large parts of the nodule, with the exception of the meristematic region and the nodule center (Figs 2d, S4). These results suggest that *nifH* nodules fail to degrade the starch that is accumulated in the infection zone.

Taken together, histological analyses indicate that bacteroid differentiation in *nifH* nodules is arrested; however, many arrested cells remain alive. In addition, the accumulation of starch in *nifH* nodules suggests that the host sequesters C to cellular storage compartments, instead of supplying it to the bacteroids.

### Metabolomic analysis of sanctioning

To gain more detailed insights into the phenomenon of sanctioning, we performed a metabolomic analysis of nodules under the experimental conditions outlined previously (control, *nifH*, argon, and high N), reflecting low, medium, and high plant N status. These conditions result in either successful symbiosis (at intermediate N status) or inhibition of symbiosis (at low or high N status) (Fig. S5a), thus allowing to distinguish pathways related to N status from mechanisms related to repression of rhizobial colonization. Global clustering according to similarity of metabolomic datasets revealed a highly consistent pattern, showing that the dataset is robust and reliable and that each treatment results in a characteristic metabolomic pattern (Fig. S5b).

To distinguish metabolites related specifically to N status from metabolites related to symbiotic status, we compared treatments pairwise and in groups. We determined common patterns of metabolic response under conditions of cheating by nonfixing rhizobia (*nifH* and argon vs controls), and of more general mechanisms involved in inhibition of nodule growth (*nifH* and argon and high N vs controls). Furthermore, high N treatments served as a positive control for the metabolic consequences of a high N status (high N vs controls) in contrast to N limitation (*nifH* and argon vs control). Ultimately, this approach allowed separation of the metabolic consequences of N depletion from the mechanisms involved in sanctioning.

When analyzing metabolomics data from nodules, it must be noted that many primary metabolites (e.g. amino acids) can be produced by both partners, the plant and the rhizobium, and their respective contributions cannot be distinguished by mass spectrometry. In cases of biosynthetic pathways that occur in only one of the two partners, however, the origin of the compounds can be inferred with confidence.

Although the respective contribution of plant and rhizobium to pools of primary metabolites cannot be determined, it can be assumed that their levels in nodules are dominated by the plant, based on the fact that the transcriptome of *Medicago* nodules contains plant and rhizobial RNA reads at a proportion of *c*. 85%:15% (Roux *et al*., 2014), and assuming that RNA content is proportional to biomass. Given that RNA content tends to be inversely correlated with body size (Lopez *et al*., 2023), contribution of plant biomass in nodules may even be > 85%.

### N‐related metabolic patterns in response to various N states

First, the most‐discriminant metabolites were determined in volcano plots comparing the sets of control nodules vs nonfixing nodules (argon and *nifH*) (Fig. S6). In order to identify N‐fixation‐related effects, only metabolites behaving similar in both, *nifH* and argon treatments, were considered. At this level of the analysis, chemical features were identified by automatic annotation, which sometimes resulted in several mentionings of the same substance (e.g. Gln, Asn, Lys; Fig. S6), indicative of isomers that have similar masses, but distinct separation kinetics in the HPLC column. Thus, the identity of metabolites of interest was subsequently verified with standards (will be discussed later). Consistent with their assumed N status (Fig. S5a), control nodules were enriched, relative to nonfixing nodules, in several N‐rich intermediates, such as Gln, Asn, and guanine, besides several other purine‐related derivatives, for example methylguanine (Fig. S6).

In the comparison of control nodules to those from high‐N plants, two groups of molecules were particularly discriminative: in high‐N samples, several N‐rich compounds were even higher than in the controls; and in control nodules, several molecules were detected with quaternary N atoms, also known as betaines (McNeil *et al*., 1999). Additional betaine‐like metabolites induced in control nodules were stachydrine (N‐dimethylproline) and trigonelline (N‐methylnicotinic acid), together with their respective precursors: pro, choline, and nicotinamide (Fig. S7). Betaines generally accumulate in response to osmotic stress and can serve as osmoprotectants (Vriezen *et al*., 2007). Stachydrine is essential for nodulation, since bacterial *stc* mutants, which cannot catabolize stachydrine, are symbiosis‐defective (Goldmann *et al*., 1994). While glycine betaine can be synthesized by both partners (Gorham, 1995; Mandon *et al*., 2003), biosynthetic pathways for stachydrine and trigonelline are not known in rhizobia, whereas their respective uptake and catabolic pathways are well‐established (Goldmann *et al*., 1991, 1994; Phillips *et al*., 1998; Alloing *et al*., 2006). Hence, stachydrine and trigonelline are likely of plant origin.

Particularly, discriminant molecular features in high‐N treatments were ureides such as allantoin, allantoic acid, and ureidoglycine (Fig. S7). Ureides represent the predominant transport form of fixed N in legumes from tropical areas such as soybean (Todd *et al*., 2006), whereas *M*. *truncatula* is considered an ‘amidic’ species that transports most of its fixed N in the form of Asn (Fischinger & Schulze, 2010). Interestingly, control nodules accumulated guanine and hypoxanthine (Fig. S7), the precursors of xanthine, allantoin, and allantoic acid that were found in high‐N treatments (Figs S7, S8). Allantoin and allantoate are not known to be produced by bacteria, unless they are genetically engineered for that purpose (Zhang *et al*., 2022), and the ureide pathway is a central feature of symbiotic legumes (Tajima *et al*., 2004; Todd *et al*., 2006); hence, allantoin and allantoic acid are likely to be of plant origin.

**Fig. 3:**
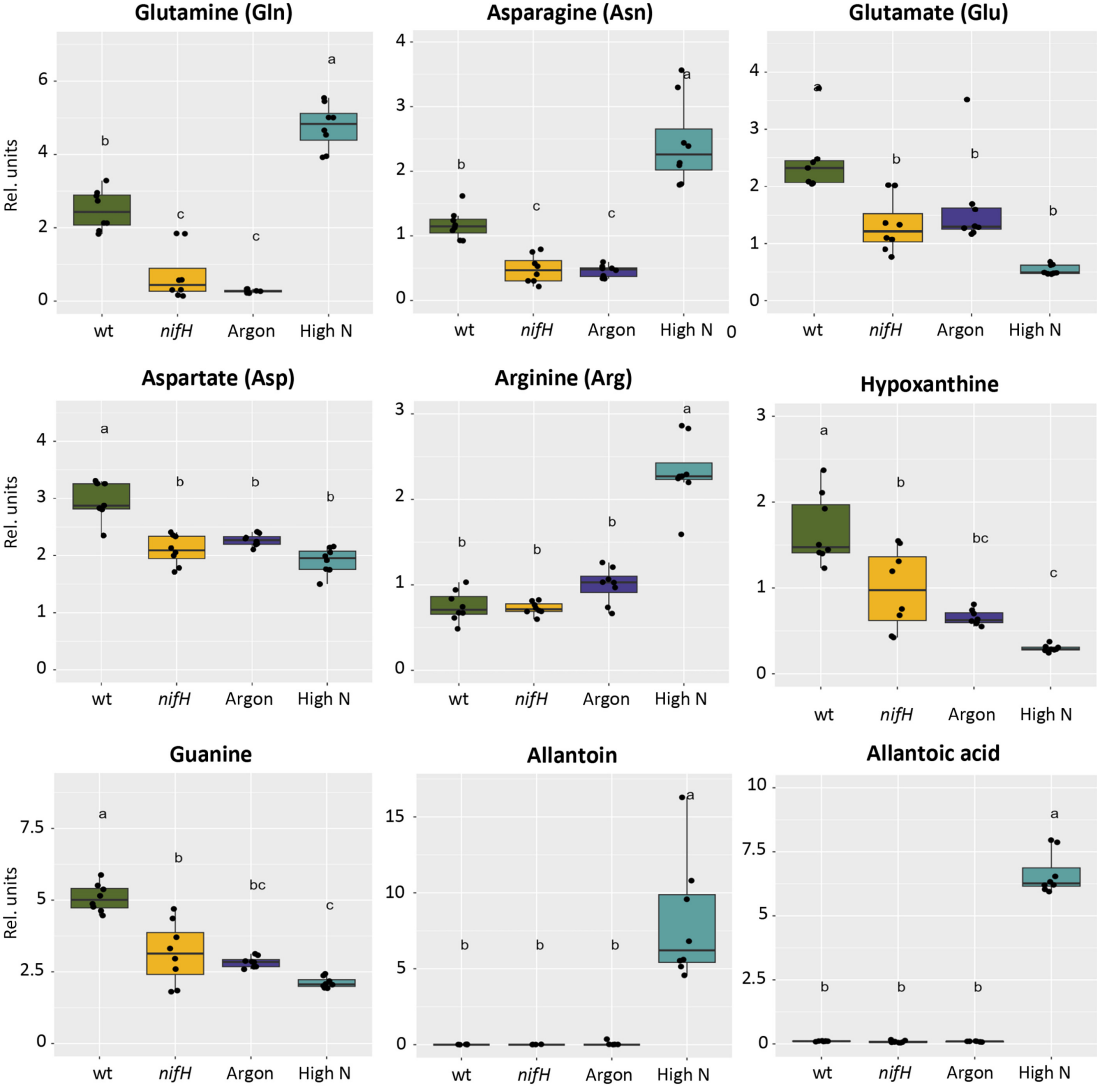
Levels of diagnostic amino acids, purines, and ureides during sanctioning in *Medicago truncatula* nodules. The relative levels of central amino acids in N assimilation, Gln and Asn, and their precursors Glu and Asp, as well as the N‐rich amino acid Arg are shown. Furthermore, the contents of the purines hypoxanthine and guanine, and of the ureides allantoin and allantoic acid are shown. Boxplots represent the median and the interquartile range; whiskers indicate the data range excluding outliers. Significance of differences was tested with one‐way ANOVA followed by Tukey's *post hoc* test (*n* = 8). Letters above box plots indicate statistically significant differences between treatments.

In order to identify and quantify molecular features with certainty, standards were run for each metabolite of interest. Quantitative determination of the N assimilation intermediates Gln and Asn (Xu *et al*., 2012) showed that controls indeed contained intermediate amounts, whereas they were low in *nifH* and argon samples, as observed previously for Gln in *nifH* nodules (Barsch *et al*., 2006), and increased in high‐N treatments (Fig. 3). Glu and Asp levels were decreased in all treatments relative to the controls, in particular under high‐N conditions, presumably reflecting depletion due to strong glutamine synthetase (GS) and asparagine synthetase (AS) activity that convert Glu to Gln and Asp to Asn, respectively (Xu *et al*., 2012). The levels of the high‐N amino acid Arg (6 C atoms and 4 N atoms per molecule) were also consistent with elevated N levels in high‐N treatments (Fig. 3).

### Sanctioning affects purine and carbohydrate metabolism

Quantitative assessment of purines and their catabolites revealed high levels of hypoxanthine and guanine in control nodules, whereas they decreased in all other treatments (Fig. 3). On the other hand, the purine catabolites allantoin and allantoic acid, which are normally not detected in nodules of *M. truncatula*, were strongly induced under high‐N conditions (Fig. 3). This indicates a transition from purine anabolism under control conditions to purine degradation at high N (Fig. S8) (Zrenner *et al*., 2006). Alternatively, high‐N supply may induce a new pathway for N‐transport related to the ureide pathway known from tropical legumes such as soybean (Todd *et al*., 2006).

**Fig. 4:**
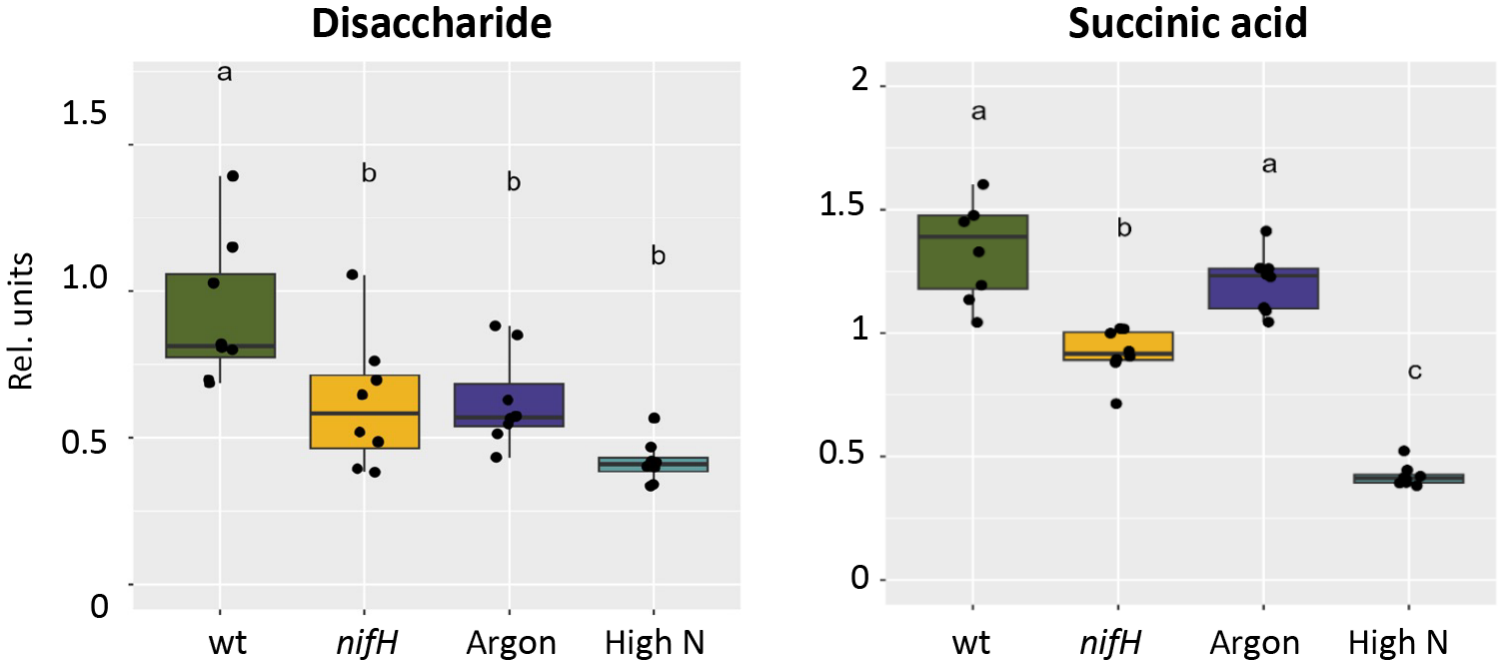
Central carbon intermediates relevant for bacteroid nutrition in *Medicago truncatula* nodules. The contents of disaccharides (sucrose and isomers such as maltose) and of the dicarboxylic acid succinic acid are shown. Boxplots represent the median and the interquartile range; whiskers indicate the data range excluding outliers. Significance of differences was tested with one‐way ANOVA followed by Tukey's *post hoc* test (*n* = 8). Letters above box plots indicate statistically significant differences between treatments.

Sugars are central metabolites and C‐transport intermediates from the shoot to roots and nodules, while rhizobial C supply is dominated by dicarboxylic acids (Yurgel & Kahn, 2004; Udvardi & Poole, 2013). Because disaccharides such as sucrose and maltose have identical molecular masses, they could not be distinguished and were therefore treated here collectively as disaccharides. The levels of both, disaccharides and succinic acid, decreased in all sanctioning conditions, although the difference for succinic acid in the argon sample was not significant (Fig. 4). Regardless of the origin of the disaccharide and succinic acid, from plant or rhizobia, lower levels suggest that carbon supply to bacteroids is likely to be reduced in sanctioned nodules. Reduced C supply was also observed in sanctioned pea nodules, even before nodule size was affected (Westhoek *et al*., 2021).

### Sanctioning decreases levels of osmoprotective metabolites

**Fig. 5:**
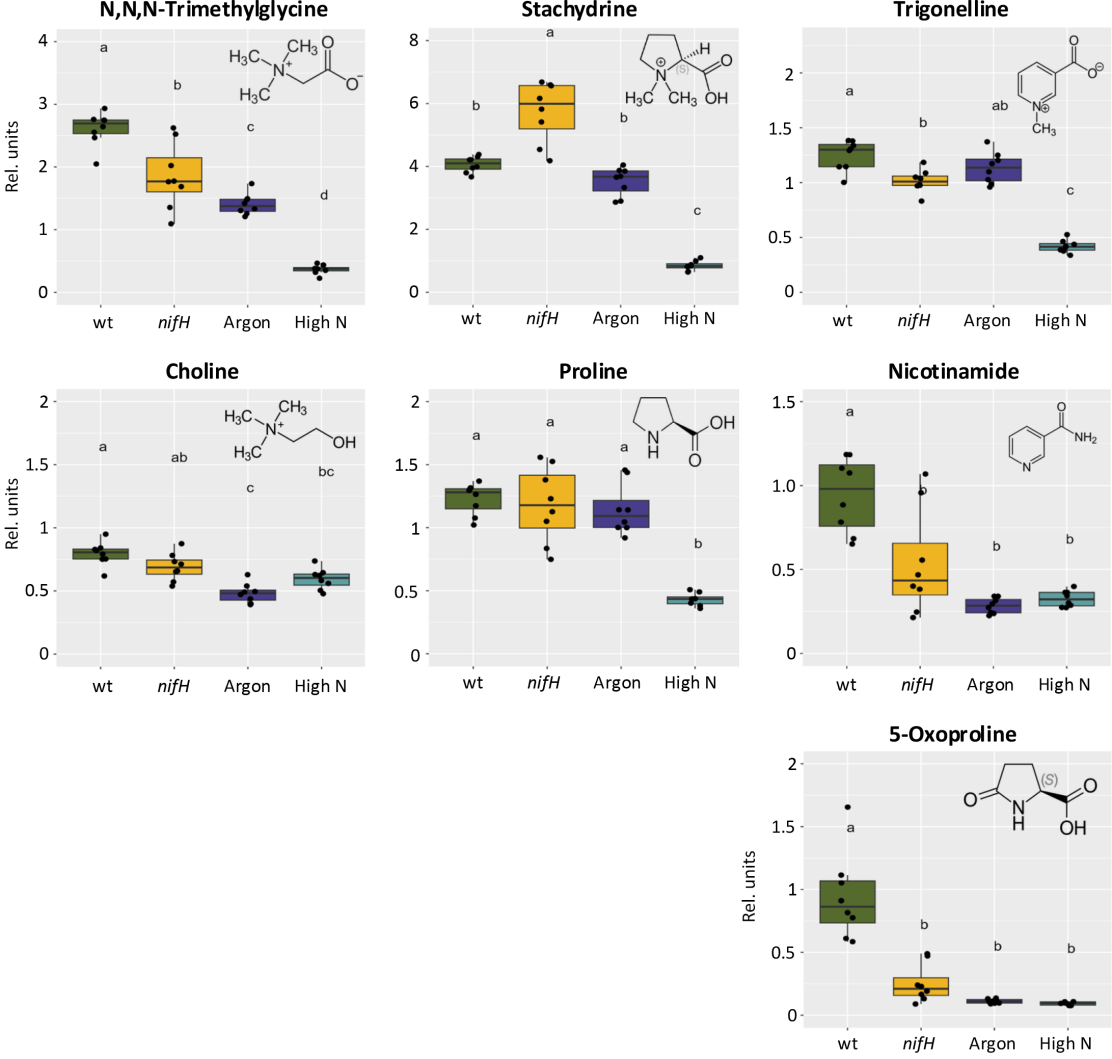
Contents of betaines and their precursors during sanctioning in *Medicago truncatula* nodules. The relative levels of glycinebetaine (N,N,N‐trimethylglycine) and its precursor choline, stachydrine and its precursor Pro, as well as trigonelline and nicotinamide, are shown, together with an additional compatible solute, 5‐oxoproline (also known as pyroglutamate). Boxplots represent the median and the interquartile range; whiskers indicate the data range excluding outliers. The significance of differences was tested with one‐way ANOVA followed by Tukey's *post hoc* test (*n* = 8). Letters above box plots indicate statistically significant differences between treatments.

Since several betaines were among the most‐discriminant molecular features (Figs S6, S7), we quantified them together with their respective precursors. In general, these metabolites showed a decreasing tendency under sanctioning, which, however, was not consistent and significant in all cases (Fig. 5). The most prominent downregulation was observed for the classical betaine N,N,N‐trimethylglycine (glycine betaine, GB), which is known to mediate stress tolerance in plants under various conditions (Sakamoto & Murata, 2002). Betaine levels were most prominently reduced under conditions of high‐N supply (Fig. 5). A similar pattern was observed for 5‐oxoproline (also known as pyroglutamate) (Fig. 5), which can protect plants against different abiotic stresses, and which is an intermediate in glutathione metabolism (Paulose *et al*., 2013; Dorion *et al*., 2021; Lei *et al*., 2024). Taken together, these results indicate that sanctioning is associated with changes in C allocation to the bacterial partner and with a decrease in stress protection with lower levels of compatible solutes in nodules.

### Global proteomic analysis of sanctioning

To obtain deeper insight into the physiological changes associated with sanctioning, we explored the proteome of the two partners to search for mechanisms that could be relevant for sanctioning. Based on metabolomic evidence, and inspired by information from the literature (Udvardi & Kahn, 1993; Denison, 2000), we mainly considered three complementary (mutually nonexclusive) hypotheses: (1) sanctioning is associated with nutritional shifts (e.g. lower C supply to rhizobia); (2) sanctioning is accompanied by repression of essential symbiotic mechanisms; and (3) sanctioning involves the induction of a host defense response against bacterial cheaters.

We generated two independent proteomic datasets (Datasets 1 and 2). Dataset 1 involved first a comparison between nodules and noninoculated roots and between nodules and free‐living rhizobia, in order to establish the symbiosis‐specific responses in the two partners. In addition, Dataset 1 contained comparisons between plants that were inoculated with either wt *S*. *meliloti* (in regular atmosphere), *nifH* mutant (in regular atmosphere), and wt *S*. *meliloti* (in argon atmosphere) (as discussed earlier) for 3 wk. Dataset 2 involved the same treatments in addition to a high‐N treatment; however, in this case, the treatments (argon, high N) were applied for only 7 d after 2 wk under control conditions, as performed for the metabolomics experiment described previously. Dataset 1 was generated with a label‐free protocol, whereas Dataset 2 involved TMT labeling.

Global clustering and Gene Ontology (GO) term analysis for functions significantly affected by both sanctioning treatments (*nifH* and argon) vs control nodules in the 3‐wk treatment (Dataset 1) revealed that symbiosis‐related pathways were downregulated (Cluster 551) and many defense‐ and stress‐related protein classes were induced (Cluster 555) in sanctioned nodules (Fig. S9; Table S1). Similarly, in Dataset 2, GO terms significantly affected by all three sanctioning treatments (*nifH*, argon, high N) vs control nodules included downregulated symbiotic functions (Cluster 1085), and induced protein groups related to oxidative stress and defense (Cluster 1091) (Fig. S10; Table S2). These results show that sanctioning treatments cause reproducible changes in proteomic patterns. Mapping induced defense markers on the Kyoto Encyclopedia of Genes and Genomes map ‘defense’ showed that induction occurred at different levels of defense (Fig. S11).

To corroborate this proteomic pattern, we performed volcano plot analysis with genes in the GO term ‘defense’ and with the symbiosis‐related NCR peptides (NCRs) (Downie & Kondorosi, 2021). Comparison of nodules with noninoculated roots revealed that NCRs are mostly nodule‐specific (only one NCR was detected in noninoculated roots) and that roots exhibit a higher defense status than nodules (Fig. S12a), in agreement with the notion that establishment of symbiosis involves repression of defense pathways (Benezech *et al*., 2020; Berrabah *et al*., 2024). By contrast, defense markers were induced in nodules colonized by *nifH*, and NCR peptide levels were decreased (Fig. S12b). A similar pattern was observed in nodules grown under continuous argon atmosphere (Fig. S12c). The treatments with argon and high N for 7 d, and the repeated inoculation with *nifH* (Dataset 2), resulted in comparable patterns of regulation (Fig. S12d–f). Taken together, these results show that sanctioning is associated with increased defense and decreased symbiotic functions.

### The plant proteome under sanctioning indicates loss of symbiotic features and induction of defense

Next, we tested the hypothesis that sanctioning involves a change in global regulatory pathways away from symbiosis. First, Datasets 1 and 2 were combined in two separate master files for the plant and the rhizobium, respectively (Tables S3, S4), which were then screened for proteins with informative patterns of regulation and for functionally defined groups of proteins. Since sanctioning from the host appears to result in an ‘anti‐symbiotic’ state, we first looked for proteins that showed a reversed pattern of regulation under sanctioning compared with nodules, for example, induced in nodules vs roots but repressed under sanctioning relative to control nodules, or vice versa, repressed in nodules vs roots but induced under sanctioning relative to control nodules (Excel sheets ‘nod‐up_sanc‐down’ and ‘nod‐down_sanc‐up’, respectively, in Table S3). These sheets were created by first establishing the condition up or down in nodules vs roots and then filtering for proteins that had the respective opposite regulation in at least three of the five sanctioning conditions (Datasets 1 and 2) (Table S3). Among 176 nodule‐specific or nodule‐induced plant proteins that were downregulated during sanctioning, we observed many NCRs and Lbs (sheet ‘nod‐up_sanc‐down’ in Table S3). Conversely, 117 proteins that were repressed in nodules showed increased levels during sanctioning, including multiple receptors, pathogenesis‐related (PR) proteins, peroxidases, glutathione‐*S*‐transferases (GSTs), and cell wall‐related proteins (sheet ‘nod‐down_sanc‐up’ in Table S3).

Based on these findings, we filtered the complete protein lists for functionally relevant protein groups using search terms such as ‘leghemoglobin’, ‘NCR’, ‘Lignin’, ‘LRR’, ‘GST’, and ‘PR‐protein’ (see respective sheets in Table S3). As expected from the lack of pink pigmentation in sanctioned nodules (Fig. 1c), the symbiosis‐induced (and two nodule‐specific) Lbs were generally repressed under sanctioning conditions (Table S3, sheet ‘Lb’; see also sheet Table S3 ‘Lb nomenclature’, for information on Lb identity according to Larrainzar *et al*., 2020), and the nodulation‐specific NCRs were also generally downregulated (Table S3, sheet ‘NCR’).

Conversely, many defense‐related protein groups were generally repressed in nodules compared with roots, and induced during sanctioning compared with control nodules. These defense‐related proteins included lignin biosynthetic enzymes (Table S3, sheet ‘Lignin’), Leucine‐rich repeat (LRR) proteins, GSTs, PR proteins, antimicrobial enzymes, among which many are also classified as PR proteins (see respective sheets in Table S3), other defense‐related proteins identified by GO term annotation ‘biological process/defense’ (Table S3, sheet ‘Defense GOBP’), or gene groups isolated by individual searches with defense‐related keywords (Table S3, sheet ‘defense_others’). Furthermore, senescence‐related proteins, including the well‐characterized nodule‐senescence markers CP6 and VPE (Pierre *et al*., 2014), were generally induced as a consequence of sanctioning (Table S3, sheet ‘senescence’). These results show that sanctioning entails a generally induced defense status and accelerated senescence in the host plant, while symbiotic functions are repressed.

### Sanctioned rhizobia suppress symbiotic markers and induce proteins associated with a free‐living lifestyle

Considering the bacterial proteome, we adopted the same logic by identifying symbiosis‐specific or symbiosis‐induced proteins that are repressed during sanctioning (Table S4, sheet ‘nod‐up_sanc‐down’), and, conversely, proteins that are repressed in nodules but induced by sanctioning (Table S4, sheet ‘nod‐down_sanc‐up’). This analysis revealed that many nodule‐specific and nodule‐induced genes were downregulated in sanctioned nodules relative to control nodules, including many essential proteins for symbiosis, such as the nitrogenase subunits NifK, NifD, NifH, several Fix proteins, the dicarboxylate transporter DctA, the NCR transporter BacA, and many proteins involved in central C metabolism (Table S4, sheet ‘nod‐up_sanc‐down’). Proteins with the opposite pattern of regulation included a diverse range of proteins, in particular sugar transporters (Table S4, sheet ‘nod‐down_sanc‐up’).

A search for functionally defined protein groups emerging from the nontargeted analysis revealed that most enzymes of the tricarboxylic acid (TCA) cycle were induced in bacteroids compared with free‐living bacteria (Table S4, sheet ‘TCA cycle’). The TCA cycle is essential to produce the large amounts of energy required for N‐fixation and for production of precursors in various biosynthetic pathways (Driscoll & Finan, 1993; Dunn, 1998; Grzemski *et al*., 2005; Udvardi & Poole, 2013; Geddes & Oresnik, 2014), although it appears that its decarboxylating branch (between citrate and succinate) is downregulated in bacteroids compared with free‐living bacteria (Schulte *et al*., 2021). Under sanctioning conditions, most TCA‐related enzymes were downregulated, in particular in Dataset 2 (Table S4, sheet ‘TCA cycle’). The subunits of the multimeric pyruvate dehydrogenase (PDH) complex (PdhA,B,C), which produces acetyl‐CoA as a substrate for citrate synthase at the entry point of the TCA cycle, showed similar patterns of regulation as the core TCA enzymes (Table S4, sheet ‘TCA cycle’). A comparable regulatory pattern was observed for protective proteins identified by the search terms ‘chaperonin’ and ‘heat shock’ (Table S4, sheet ‘protect’), and for DNA‐related proteins (Table S4, sheet ‘DNA’).

Interestingly, some protein groups showed an opposite behavior. For example, sugar‐related ATP‐binding cassette (ABC) transporters tended to be downregulated in bacteroids compared with free‐living rhizobia, presumably reflecting the fact that bacterial C supply involves dicarboxylic acids rather than sugars (Yurgel & Kahn, 2004; Udvardi & Poole, 2013). Under conditions of sanctioning, however, most sugar transporters were induced, possibly indicating compensatory induction in response to C shortage (Fig. 4).

We also detected proteins involved in motility and cell division, which were downregulated in nodules compared with roots. Notably, these proteins were induced during sanctioning (Table S4, sheet ‘motility & division’). These results suggest that sanctioned bacteroids do not just undergo general reduction in protein levels, as might be expected as a consequence of premature senescence. Rather, it indicates that sanctioned rhizobia repress symbiotic functions, while markers of a free‐living life style, such as motility and cell division, are activated.

### The host phosphoproteome reveals induced defense under sanctioning

Because many plant and bacterial proteins are under posttranslational control by phosphorylation, we assessed the nodule phosphoproteome under control conditions and under the three sanctioning conditions (*nifH*, argon, and high N) in Dataset 2. Differential phosphorylation was observed in 13 683 tryptic peptides, which were separately analyzed for plant and bacterial phosphopeptides (Table S5). We ordered the entire table according to the highest relative phosphorylation levels in *nifH* vs control, and identified the top 100 plant phosphopeptides (0.7% of the total). We then retained only items significantly induced in at least two of the three sanctioning treatments and removed all hypothetical proteins. The resulting list of phosphopeptides fell into five functionally related groups (Table S5, sheet ‘Plant Phosphoproteins (Top100)’).

The first group is related to defense, including a Mitogen‐activated protein kinase kinase kinase (MAPKKK), components of Ca and oxidative signaling, and the defense regulator RIN4, which was phosphorylated on a conserved Ser residue (Ser85) (Fig. S13a). Phosphorylation of RIN4 represents a phosphoswitch that can increase (or reduce) immunity of *Arabidopsis thaliana* depending on the site of phosphorylation (Chung *et al*., 2014; Toruño *et al*., 2019). Strikingly, a recent study documented that RIN4 is required for nodule symbiosis in soybean, suggesting that it gates the defense response to mediate either symbiosis or disease resistance (Toth *et al*., 2023). In *M. truncatula*, a total of 15 phosphopeptides of RIN4 were detected (Fig. S13; Table S5), with one phosphosite (Ser85, red in Fig. S13a) being among the 100 most induced phosphorylation events (Table S5; Fig. S13b). Phosphorylation of the corresponding conserved residue in *A. thaliana* confers disease resistance (Liu *et al*., 2011), suggesting that Ser85 phosphorylation in *M. truncatula* may trigger defense. A dephosphorylated site was detected at Ser140 (blue in Fig. S13a), while a symbiosis‐related phosphorylation site in a conserved legume‐specific motif (GRDSP) (Toth *et al*., 2023) showed intermediate phosphorylation (green in Fig. S13a). RIN4 in soybean interacts with the symbiosis receptors nod factor receptor 1 (NFR1) and symbiosis receptor‐like kinase (SYMRK) and is phosphorylated by the latter in the GRDSP‐motif (Ser143 in soybean; Ser158 in *M. truncatula*) (Toth *et al*., 2023).

The second group contained essential elements of nodule functioning, such as Lbs and an NCR. Lb phosphosites Ser13, Ser14 in helix A, and Ser50 and Ser56 in a hinge loop between helix D and E are shared among six of the 12 Lb proteins of *M. truncatula* (Fig. S14a,b). Interestingly, phosphosite Ser92 of Lb11 is situated just 6 Å from His94, which is part of the heme coordination center (Fig. S14c); hence, it may influence O_2_ binding kinetics or protein stability. Lb phosphorylation has been observed earlier in *M. truncatula* (Marx *et al*., 2016) and other legumes (*Lotus japonicus*, soybean; data not shown); however, its functional significance is unknown.

The third group contained regulators of cellular dynamics, including proteins involved in secretion, membrane transport, membrane dynamics, and cytoskeletal elements. Notably, several phosphopeptides belonged to a nodule‐specific remorin (H2EST4; Table S5, compare with Table S3), the ortholog of which is required for symbiosis in *L. japonicus* (Raffaele *et al*., 2007; Lefebvre *et al*., 2010; Marín & Ott, 2012; Liang *et al*., 2018). Phosphorylations of *M. truncatula* SymREM1 observed during sanctioning occurred in the intrinsically disordered N‐terminal part (Fig. S15), which is also phosphorylated by the symbiotic receptor kinases NFR1 and SYMRK in *L*. *japonicus* (Marín & Ott, 2012). The phosphorylated residue Ser48 of *M. truncatula* is conserved in *L. japonicus* (Fig. S15), corresponding in this case to Ser44 that is phosphorylated *in vitro* by NFR1 and SYMRK (Toth *et al*., 2012). In *M*. *truncatula*, SymREM1 controls stability and turnover of the symbiosis receptor LYK3 at the plasma membrane and is required for symbiotic signaling during infection (Lefebvre *et al*., 2010).

The fourth group consisted of elements from central sugar and N metabolism, which may regulate C supply to nodules and N assimilation in the host, as well as Pro biosynthesis. Finally, a fifth group contained a pair of Zn finger proteins of unknown function with six phosphopeptides that were increased by sanctioning. While the functional significance of most of these phosphorylation events remains to be established, these results are compatible with the notion that sanctioning impinges on symbiotic functions and increases the defense status of the host.

### The rhizobial phosphoproteome: nitrogenase, TCA cycle, and stress signaling

The bacterial phosphoproteome encompassed only 63 phosphopeptides in total, of which 33 were significantly induced in at least two sanctioning conditions (Table S5, sheet ‘All Rhizobial Phosphoproteins’). After sorting according to induction in *nifH*, the most prominent phosphopeptide, with significant induction in all three sanctioning treatments, was PdhA, a component of the multimeric PDH enzyme complex, of which all three subunits (PdhA, PdhB, and PdhC) are significantly downregulated by sanctioning (Table S4, sheet ‘TCA’). Interestingly, four TCA cycle‐related enzymes, Icd, GltA, SucC, and SucD, which are among the downregulated components in *S. meliloti* (Table S4, sheet ‘TCA cycle’), were significantly phosphorylated in two of the three sanctioning treatments (Table S5, sheet ‘All Rhizobial Phosphoproteins’).

Furthermore, the nitrogenase components NifH and NifE were phosphorylated during sanctioning (Table S5, sheet ‘All bacterial phosphoproteins’). The NifH protein was phosphorylated within the first 25 amino acids, that is upstream of the *Tn5* insertion site in the *nifH* mutant, thereby allowing for phosphopeptides to be generated even in the mutants. Phosphorylation of Nif proteins has been observed before (Marx *et al*., 2016), but its significance remains unknown.

Remarkably, the response regulator protein ChvI showed increased phosphorylation in all sanctioning conditions. The ChvG‐ChvI signaling system represents a global regulatory circuit conserved among the alpha‐proteobacteria (Greenwich *et al*., 2023). It is required for nodule symbiosis in legumes as well as for virulence of *Agrobacterium tumefaciens* (Greenwich *et al*., 2023). As a consequence of the activation of the ChvG‐ChvI system, ChvI undergoes phosphorylation and induces downstream genes by direct transcriptional activation (Bélanger & Charles, 2013; Ratib *et al*., 2018). ChvI mutants are sensitive to low pH, and in the case of *A. tumefaciens*, low pH in the host tissues is thought to represent a signal that activates virulence factors through the ChvG‐ChvI system (Yuan *et al*., 2008). Conceivably, ChvI phosphorylation indicates that sanctioned rhizobia are subject to hyperacidification of the peribacteroid space beyond the levels normally required for functional symbiosis (Pierre *et al*., 2013), or that they experience another sort of envelope stress that triggers ChvI phosphorylation.

Significantly decreased phosphorylation levels in at least two of the three sanctioning conditions were observed for only 13 bacterial peptides, notably including three chaperonins (GroEL3, GroEL4, and GroEL5), paralleling the general downregulation of chaperonin protein levels during sanctioning (Table S4, sheet ‘Protect.’). Chaperonins are relevant for symbiosis and for stress responses (Bittner *et al*., 2007; Ansari & Mande, 2018), and their phosphorylation has been shown to promote protein oligomerization and cellular biofilm formation, indicating a function in stress‐coping mechanisms (Arora *et al*., 2017; Ansari & Mande, 2018).

## Discussion

While partner recognition is essential for symbiosis of legumes with their rhizobial partners, it is not a reliable mechanism to ensure mutualism. This is because microbes can deceive potential hosts (Behm *et al*., 2014). For example, nonfixing bacteria can gain access to a host by acquiring signalling capacity via horizontal transfer of symbiotic plasmids from cognate rhizobia (Doin de Moura *et al*., 2020; Magne *et al*., 2024). On the other hand, effective rhizobial partners can turn into cheaters by losing N‐fixation capacity, for example through mutation of essential *nif* and *fix* genes. It has therefore been predicted that LRS would decay over evolutionary time, unless the plant host had mechanisms to assess symbiotic service from the bacterial partner and to sanction nonmutualistic cheaters (Udvardi & Kahn, 1993; Denison, 2000; Kiers & Denison, 2008; Oono *et al*., 2009; Sachs *et al*., 2018). Such mechanisms save the plant from being exploited, and avoid parasitic rhizobia to proliferate in the environment. Control by the host is particularly important in an interaction as asymmetrical as LRS, in which the host has a generation time of months to years, while the endosymbiont can divide in a matter of hours. This means that cheaters are much more likely to emerge in the rhizobial partner than in the host, and, if cheaters have a selective advantage over N‐fixers (e.g. by diverting metabolic resources from N‐fixation to proliferation), they can potentially outcompete mutualistic clones and become dominant in the host and in the surrounding environment.

As predicted, many studies have documented that host plants can select beneficial rhizobial strains relative to less beneficial strains (Daubech *et al*., 2017; Westhoek *et al*., 2017, 2021), suggesting that the host can assess symbiotic service and sanction cheaters (Kiers & Denison, 2008; Boivin & Lepetit, 2020). Such systems can work in scenarios with single inoculations (e.g. with homogenous inocula consisting of nonfixing mutants), or in mixed inoculations with bacteria of different symbiotic efficiencies (Regus *et al*., 2015, 2017; Westhoek *et al*., 2017, 2021). However, the underlying mechanisms in sanctioning have remained unclear. Reduced O_2_ and C supply have been invoked as central elements in sanctioning (Kiers *et al*., 2003; Westhoek *et al*., 2021), but their contribution to the phenomenon has not been established.

**Fig. 6:**
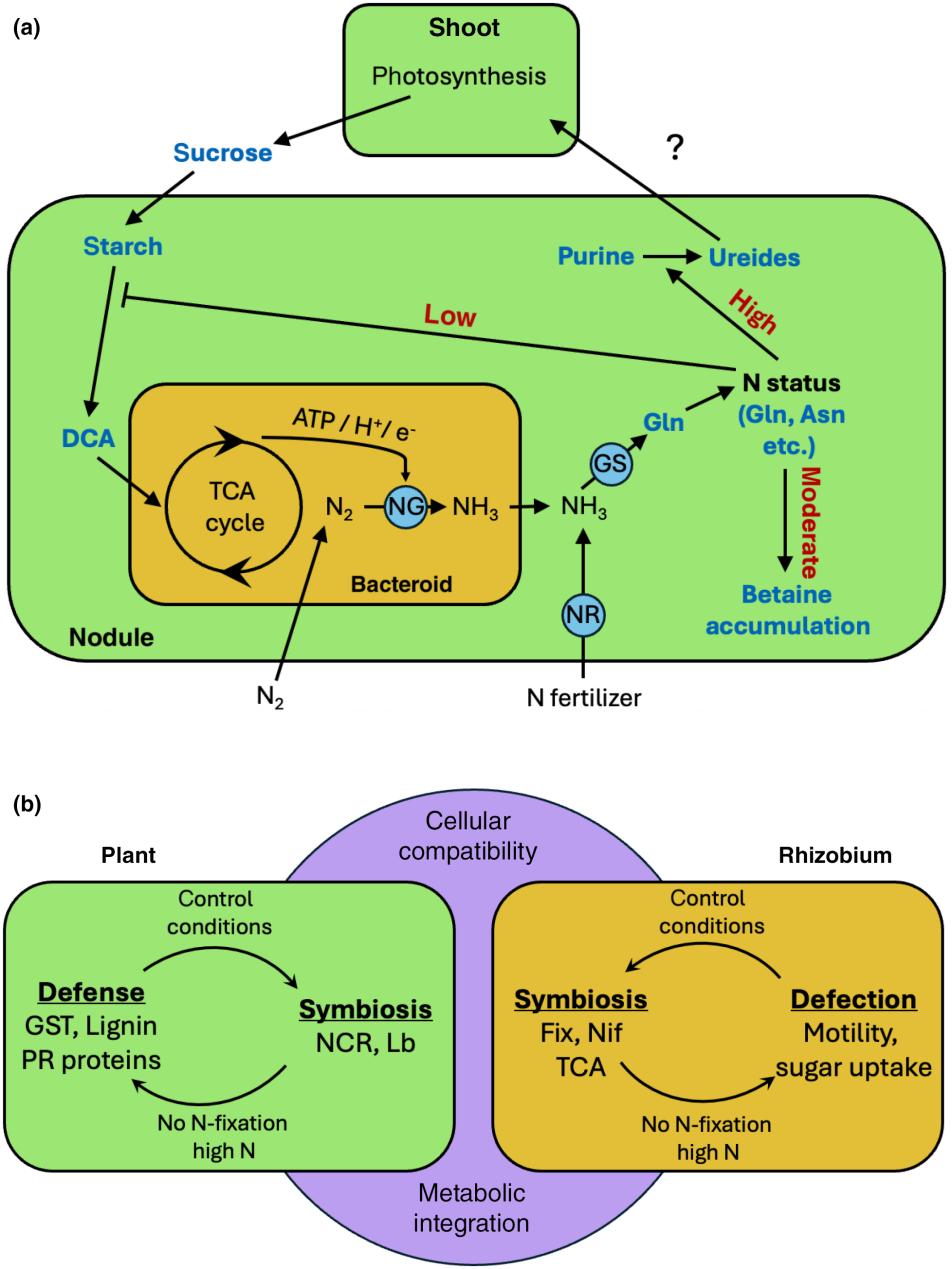
Model of mechanisms involved in sanctioning in *Medicago truncatula*. (a) Schematical representation of the fluxes of the central forms of C and N in the exchange between *M. truncatula* (green compartments) and *Sinorhizobium meliloti* (brown compartment). Carbon from photosynthesis is transferred to the nodules as carbohydrate (sucrose), intermittently stored as starch, converted to dicarboxylic acids, and delivered to the bacteroids in which it fuels the tricarboxylic acid (TCA) cycle. Reduction equivalents produced by the TCA cycle are used by nitrogenase (NG) to fix N. The primary product of NG, ammonia (NH_3_), is delivered to the host, in which it is assimilated by glutamine synthetase to Gln, which is a primary N currency that reflects N status. Sanctioning in response to nonfavorable N status inhibits carbon transfer to bacteroids. High N status triggers conversion of purines to ureides, whereas moderate N status promotes accumulation of betaines. Nitrate input from fertilizer is incorporated into the NH_3_ pool by nitrate reductase (nitrite reductase not shown). Blue font indicates molecular species that were quantified in this study. Note that some of the metabolites can be synthesized by both partners (e.g. Gln, purine). The scheme, however, represents a consensus emphasizing the central fluxes relevant for symbiosis and sanctioning, based on published genetic and omics information and on our metabolomic/proteomic data. (b) Conceptual framework of the interactions between the plant host (green) and the bacterial endosymbiont (brown) featuring the main traits and protein groups related to symbiosis (pink circle), and conferring metabolic integration and cellular compatibility, as opposed to the antisymbiotic strategies associated with sanctioning (defense in the plant; defection in the rhizobium). Control conditions allow both partners to establish symbiosis‐related pathways and move to the center, allowing for cellular compatibility and metabolic integration. By contrast, lack of N‐fixation or high N supply triggers pathways that drive both partners away from the symbiotic center toward selfish strategies.

Sanctioning of cheaters by legumes can be defined as a reduction in bacterial fitness. In principle, reduced bacterial fitness can result from inhibition of processes such as bacterial growth and proliferation. Alternatively, sanctioning could involve toxic or bactericidal mechanisms, for example through activation of a defense response. Our histological analyses of nodule development in *nifH* mutants suggests that bacterial proliferation and differentiation are inhibited in sanctioned nodules (Figs 1, 2, S1–S3). However, we did not obtain clear evidence for reduced rhizobial viability during sanctioning (Figs 2, S2). Based on our observation of starch accumulation in *nifH* nodules (Fig. 2), and on our global metabolomics and proteomics analyses, we rather suggest that a combination of reduced metabolic supply (Fig. 4), decreased stress protection (Fig. 5), and transitions in general metabolism (Figs 6a, S6–S8) restricts bacterial growth and proliferation.

In addition, inhibition of essential symbiotic traits, such as NCRs and Lbs, limits bacterial differentiation and possibly respiration (by decreasing O_2_ supply). While these effects limit bacterial growth and proliferation, they also attenuate the metabolic burden for the plant, by reducing both C supply to the bacteroids and the costly synthesis of components required for establishment and functioning of the symbiotic machinery.

In addition to metabolic adjustment, the activation of defense mechanisms during sanctioning suggests that the host directly attacks nonfixing cheaters. Induction of lignin biosynthetic enzymes and diverse defense markers (e.g. LRR proteins, PR proteins, GSTs; Table S4), as well as the phosphorylation of RIN4 (Fig. S13; Table S5), suggests that the host deploys a wide array of defense mechanisms to inhibit cheaters. However, most rhizobial cells appear to remain alive in sanctioned nodules (Figs 2, S2), conceivably as a result of induced stress‐coping mechanisms involving the ChvG/ChvI system and chaperonins. How the plant senses the lack of N fixation is unknown, but it is likely to involve sensing of the N status via intermediates of N assimilation (Berrabah *et al*., 2018).

Given the observed changes during sanctioning in the two partners, it appears that they shift from the symbiotic life style to more selfish strategies that result in their reciprocal incompatibility (Fig. 6b). In the host, this implies accumulation of starch (Figs 2, S4) at the expense of soluble C nutrients (Fig. 4) and a decline in the levels of osmoprotectants (betaines) (Fig. 5) and Lbs (Table S3), which are among the most abundant nodule proteins. In the rhizobial partner, sanctioning results in a general downregulation of the TCA cycle and an induction of sugar transporters and proteins for cell division and motility (Table S4), together with activation of stress‐coping mechanisms activated by the ChvG/ChvI system.

The fact that the interference with N fixation affects the physiology of both the host and the endosymbiont, in which the *nifH* mutation causes downregulation of many other symbiosis‐related proteins, suggests that sanctioning triggers negative feedback mechanisms in both partners, resulting in self‐reinforcing decay of symbiosis.

## Competing interests

None declared.

## Author contributions

DR conceived and supervised the project. MC, AR and NJ performed experiments. P‐MA, ED and MS performed metabolomic and proteomic analysis. IY and MB carried out protein 3D modeling and phosphoproteome analysis. DR, MC and AR wrote the manuscript.

## Disclaimer

The New Phytologist Foundation remains neutral with regard to jurisdictional claims in maps and in any institutional affiliations.

## Supporting information


**Fig. S1** Nodule development under sanctioning in *Medicago truncatula*.
**Fig. S2** Rhizobial viability and membrane permeability in *Medicago truncatula* nodules revealed by live/dead staining.
**Fig. S3** Transmission electron microscopic analysis of colonized cells with wild‐type and *nifH* bacteria in nodules of *Medicago truncatula*.
**Fig. S4** Distribution of starch in wild‐type and *nifH* nodules of *Medicago truncatula*.
**Fig. S5** Experimental design and global clustering of results from metabolomic analysis.
**Fig. S6** Global assessment of most‐discriminative chemical features of nonfixing nodules (*nifH*, argon) vs controls in *Medicago truncatula*.
**Fig. S7** Global assessment of most‐discriminative chemical features of N‐fertilized nodules in *Medicago truncatula* (high N) vs controls.
**Fig. S8** Purine *de novo* synthesis and catabolism in plants.
**Fig. S9** Global clustering of proteomics results from label‐free analysis during continuous treatments with *nifH* and argon in *Medicago truncatula* nodules (Dataset 1).
**Fig. S10** Global clustering of proteomics results from tandem mass tag‐labeled treatments with *nifH*, argon, or high N in *Medicago truncatula* nodules (Dataset 2).
**Fig. S11** Mapping of *Medicago truncatula* defense‐related proteins on the Kyoto Encyclopedia of Genes and Genomes pathway ‘Plant–pathogen interaction’.
**Fig. S12** Relative expression of nodule cysteine‐rich and defense‐related proteins in nodules of *Medicago truncatula*.
**Fig. S13** Phosphorylation pattern of *Medicago truncatula* RIN4 during sanctioning.
**Fig. S14** Analysis of leghemoglobin phosphorylation patterns during sanctioning in *Medicago truncatula*.
**Fig. S15** Phosphorylation patterns of *Medicago truncatula* SymREM1 during sanctioning.
**Methods S1** Supplementary Methods.

**Table S1** Gene Ontology term analysis of differentially regulated items significantly affected by sanctioning in *Medicago truncatula* nodules (Dataset 1).

**Table S2** Gene Ontology term analysis of differentially regulated items significantly affected by sanctioning in *Medicago truncatula* nodules (Dataset 2).

**Table S3** Plant proteome analysis under sanctioning in *Medicago truncatula* nodules.

**Table S4** Rhizobial proteome analysis under sanctioning in *Medicago truncatula* nodules.

**Table S5** Phosphoproteome analysis under sanctioning in *Medicago truncatula* nodules.
Please note: Wiley is not responsible for the content or functionality of any Supporting Information supplied by the authors. Any queries (other than missing material) should be directed to the *New Phytologist* Central Office.

## Data Availability

All primary raw data are available on public repositories. For metabolomic analysis, the full spectral dataset corresponding to the extract collection has been uploaded on the MassIVE repository https://doi.org/doi:10.25345/C57P8TR88, and a metabolite annotation workflow was automatically carried out against the Global Natural Products Social Molecular Networking (GNPS) experimental spectral libraries: https://gnps.ucsd.edu/ProteoSAFe/result.jsp?task=74a7bc6828294e8c95bdba312f1b58b7&view=group_by_compound (positive ionization mode) and https://gnps2.org/result?task=a10155676345444dba86da0964c1f43f&viewname=librarymatches&resultdisplay_type=task (negative ionization mode). The mass spectrometry proteomics data have been deposited to the ProteomeXchange Consortium via the PRIDE partner repository (Perez‐Riverol *et al*., 2024) with the dataset identifiers PXD065143, PXD065225, and PXD065245.
