# Supplementary material for: Inhibition of rhizobial cheaters by the host Medicago truncatula involves repression of symbiotic functions and induction of defense
Source: New Phytol. 2025 Sep 5;248(3):1458–74. doi: 10.1111/nph.70494 (PMC12489286; doi:10.1111/nph.70494)
Supplement: Supplementary file 1 — Fig. S1 Nodule development under sanctioning in Medicago truncatula. Fig. S2 Rhizobial viability and membrane permeability in Medicago truncatula nodules revealed by live/dead staining. Fig. S3 Transmission electron microscopic analysis of colonized cells with wild‐type and nifH bacteria in nodules of Medicago truncatula. Fig. S4 Distribution of starch in wild‐type and nifH nodules of Medicago truncatula. Fig. S5 Experimental design and global clustering of results from metabolomic analysis. Fig. S6 Global assessment of most‐discriminative chemical features of nonfixing nodules (nifH, argon) vs controls in Medicago truncatula. Fig. S7 Global assessment of most‐discriminative chemical features of N‐fertilized nodules in Medicago truncatula (high N) vs controls. Fig. S8 Purine de novo synthesis and catabolism in plants. Fig. S9 Global clustering of proteomics results from label‐free analysis during continuous treatments with nifH and argon in Medicago truncatula nodules (Dataset 1). Fig. S10 Global clustering of proteomics results from tandem mass tag‐labeled treatments with nifH, argon, or high N in Medicago truncatula nodules (Dataset 2). Fig. S11 Mapping of Medicago truncatula defense‐related proteins on the Kyoto Encyclopedia of Genes and Genomes pathway ‘Plant–pathogen interaction’. Fig. S12 Relative expression of nodule cysteine‐rich and defense‐related proteins in nodules of Medicago truncatula. Fig. S13 Phosphorylation pattern of Medicago truncatula RIN4 during sanctioning. Fig. S14 Analysis of leghemoglobin phosphorylation patterns during sanctioning in Medicago truncatula. Fig. S15 Phosphorylation patterns of Medicago truncatula SymREM1 during sanctioning. Methods S1 Supplementary Methods. [file NPH-248-1458-s004.pdf]

New Phytologist **Supporting Information**

Article title: **Inhibition of rhizobial cheaters by the host *Medicago truncatula* involves repression of symbiotic functions and induction of defense**

Authors: Min Chen, Axelle Raisin, Natalie Judkins, Pierre-Marie Allard, Emmanuel Défossez, Michael Stumpe, Inmaculada Yruela, Manuel Becana, Didier Reinhardt

Article acceptance date: 29 July 2025

The following Supporting Information is available for this article:

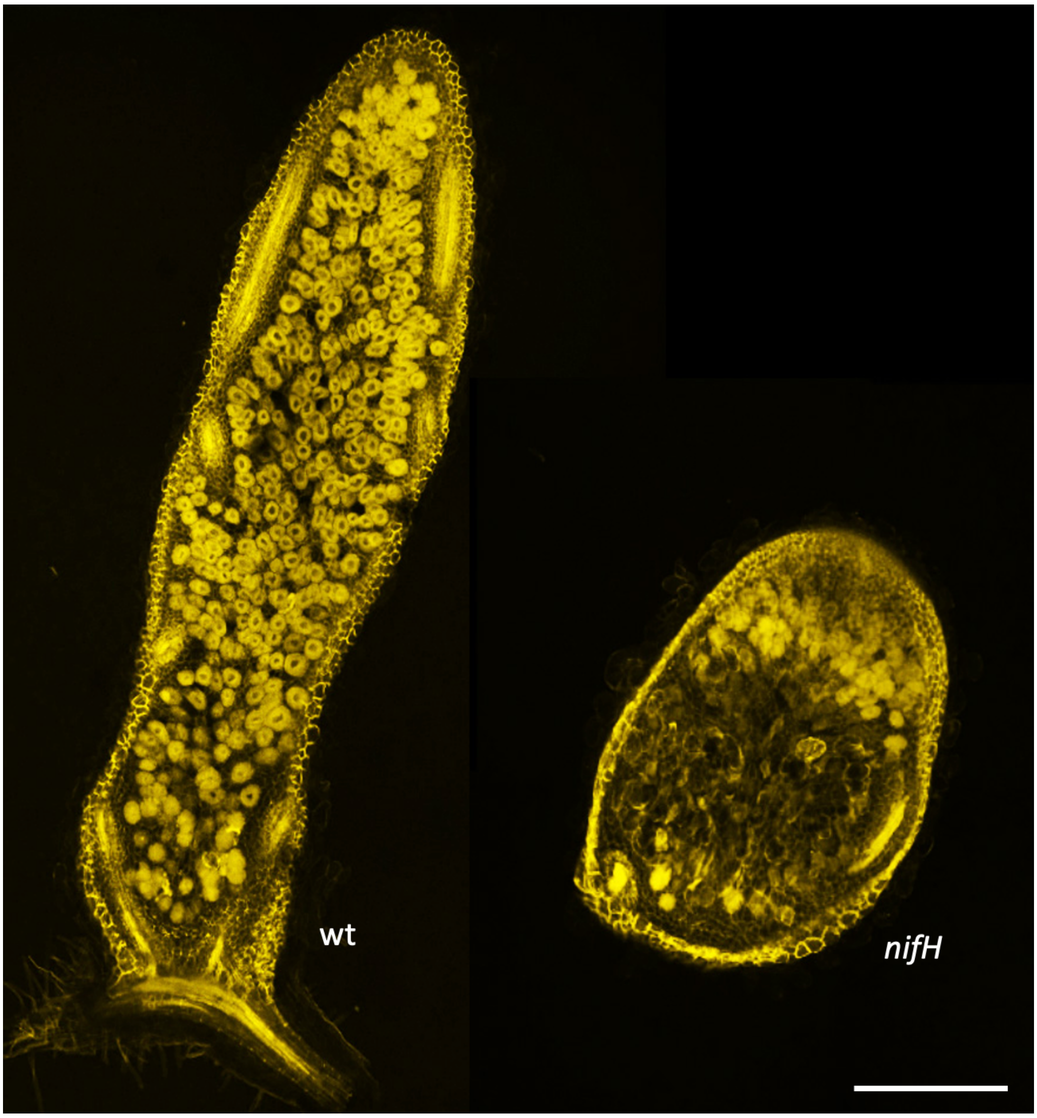

Fig. S1 Nodule development under sanctioning in *M. truncatula*

Anatomy of nodules inhabited by wild-type rhizobia (wt) and *nifH* mutants (*nifH*) cultured for 35 d under control conditions. Shown are confocal images after staining with basic fuchsin. Size bar 0.5 mm.

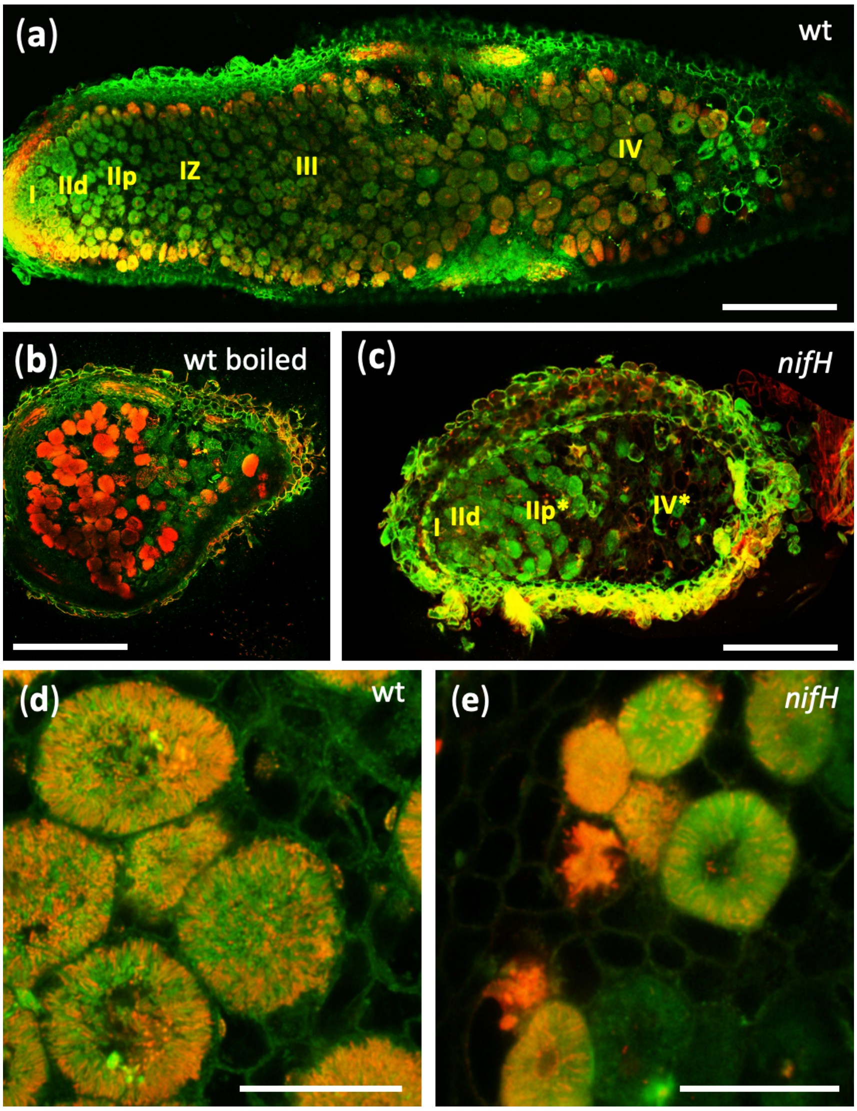

Fig. S2 Rhizobial viability and membrane permeability in *M. truncatula* nodules revealed by live/dead staining.

(**a**) Nodule inhabited by wild-type rhizobia after 35 d from inoculation. Vibratome sections were stained with SYTO9 (green) and propidium iodide (PI; red), revealing partial membrane permeability (weak reddish staining) of bacteroids. The developmental gradient of bacteroid differentiation is indicated with the approximate location of the meristematic zone (I), distal and proximal infection zone (IId, IIp), the interzone (IZ), the fixation zone (III), and the senescence zone (IV). (**b**) A nodule as in (**a**) incubated for 5 min in boiling water. Strong red staining indicates dead cells with completely permeable cell membranes. (**c**) Nodule as in (**a**), but inhabited by *nifH* mutant bacteria after 35 d from inoculation. Note aberrant zonation with a deviating zone IIp* which tapers of into the sparsely colonized basal region of the nodule that resembles the senescence zone (IV*). The fixation zone (ZIII) is essentially missing. (**d,e**) Individual infected cells inhabited by wild-type bacteroids (**d**), and inhabited by *nifH* mutants (**e**). Size bars 250 µm in **a**, **b**, **c**; 50 µm in **d**, **e**.

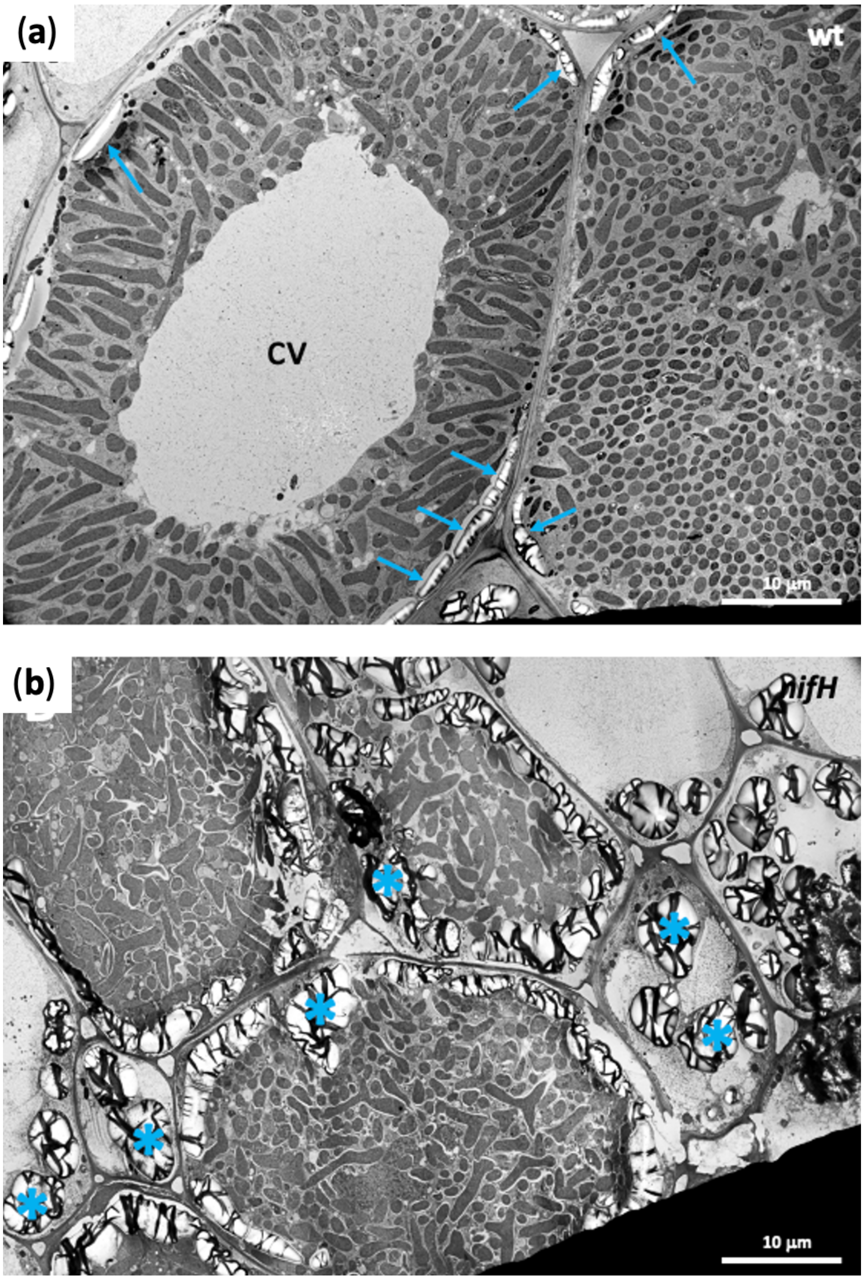

Fig. S3 Transmission electron microscopic analysis of colonized cells with wild-type and *nifH* bacteria in nodules of *M. truncatula*

(**a**) Two neighboring colonized nodule cells with fully differentiated bacteroids that fill most of the cytoplasm around the central vacuole (cv). Note the general alignement of bacteroids reflected by mostly longitudinal sections in the left cell, and predominatly transverse sections in the right cell. (**b**) Three neighboring colonized nodule cells with aberrant *nifH* bacteroids. None of the three cells shows a central vacuole, and the bacteroids appear to be oriented randomly in all directions. Note large amyloplasts (light objects with dark stripes) along the cellular periphery (asterisks). Corresponding amyloplasts in colonized cells of wild-type nodules are narrow and tend to be aligned with the cell wall (arrows in **a**). Size bars 10 µm

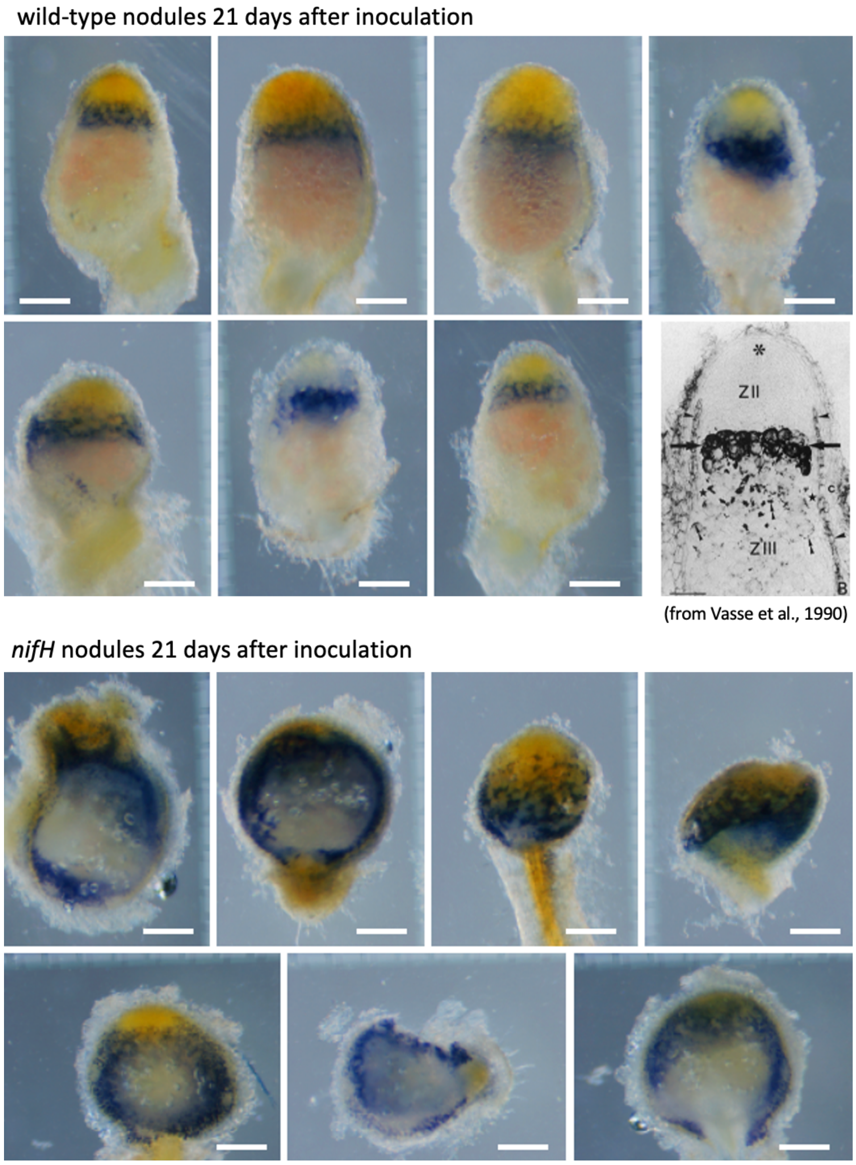

Fig. S4 Distribution of starch in wild-type and *nifH* nodules of *M. truncatula*.

Three-wk-old nodules were stained with lugol for 4 min. and hand-sectioned in the longitudinal direction. For comparison, Figure 1b from Vasse *et al*., (1990) is shown exhibiting starch distribution in an alfalfa nodule. Note a restricted band of starch (purple) between the meristematic region (yellow) and the fixation zone (pink) in the wild type, whereas *nifH* nodules exhibit starch around large parts of the nodules except for the meristematic region and the center. Note the virtual absence of the pink Lb pigment from *nifH* nodules. Size bars, 250 µm.

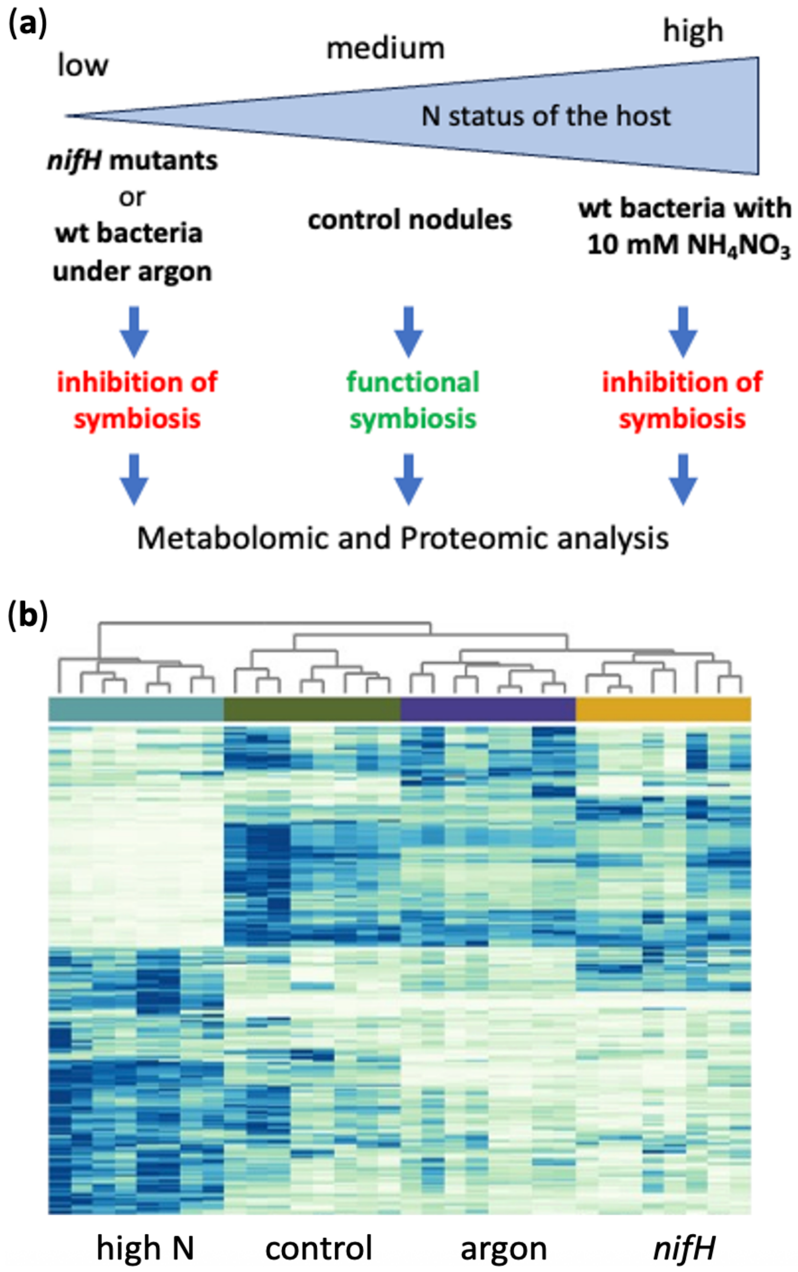

Fig. S5 Experimental design and global clustering of results from metabolomic analysis in *M. truncatula*.

(**a**) Sanctioning of the bacterial partner by the host is controlled via manipulation of N status. The symbiotic system is either deprived of N (inoculation with *nifH* mutants or exposure to argon atmosphere) or treated with high N (10 mM NH_4NO_3). In all three cases, symbiosis was aborted compared to control conditions. Nodules harvested from the four growth conditions were used for metabolomic and proteomic analyses. (**b**) Global clustering of the metabolomic dataset revealed consistent abundance patterns of chemical features. All sample types clustered according to their group. A strong shift in abundance is particularly obvious for the high N treatment.

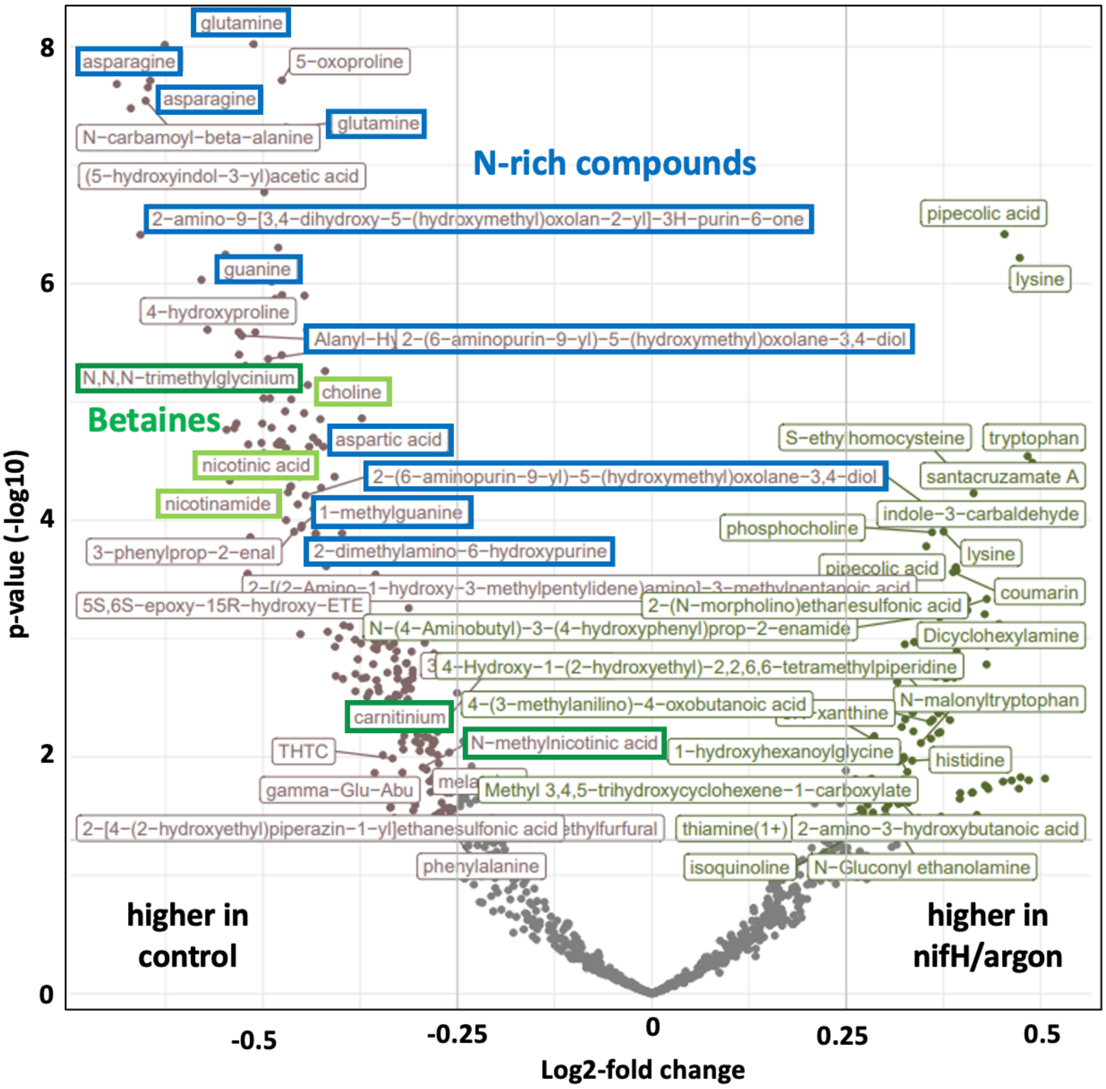

Fig. S6 Global assessment of most-discriminative chemical features of non-fixing nodules (*nifH*, argon) vs. controls in *M. truncatula*.

Volcano plot analysis of all chemical features according to the log2-fold change of non-fixing vs. controls (*x*-axis) and the -log10 *p*-value for the significance of the change (*y*-axis). Features identified by automatic annotation are indicated. Boxed compounds in color indicate betaine-like compounds (green) and their precursors (light green), and N-rich compounds (blue).

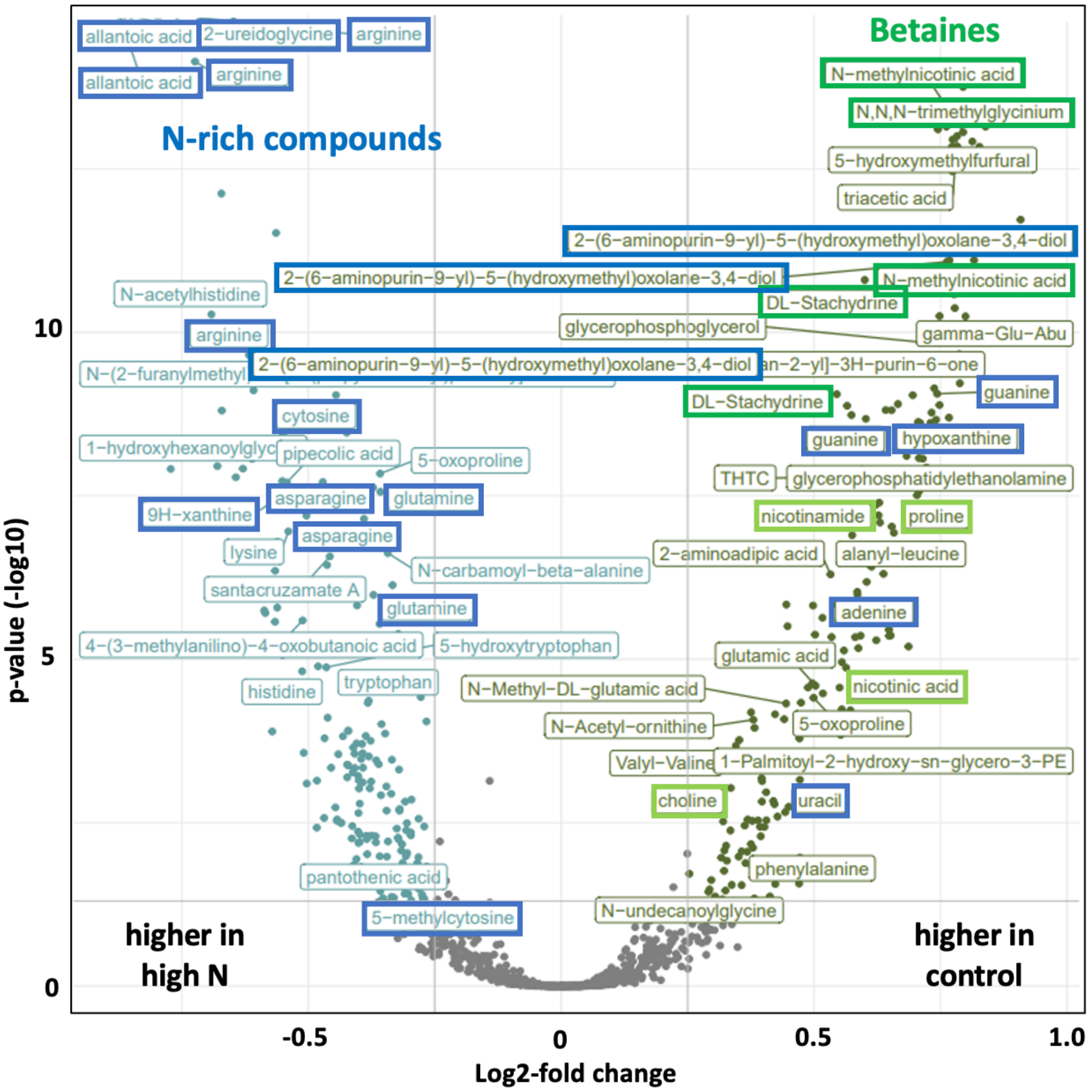

Fig. S7 Global assessment of most-discriminative chemical features of N-fertilized nodules (high N) vs. controls in *M. truncatula*.

Volcano plot analysis of all chemical features according to the log2-fold change of high N treatments vs. controls (*x*-axis) and the -log10 *p*-value for the significance of the change (*y*-axis). Features identified by automatic annotation are indicated. Boxed compounds in color indicate betaine-like compounds (green) and their precursors (light green), and N-rich compounds (blue).

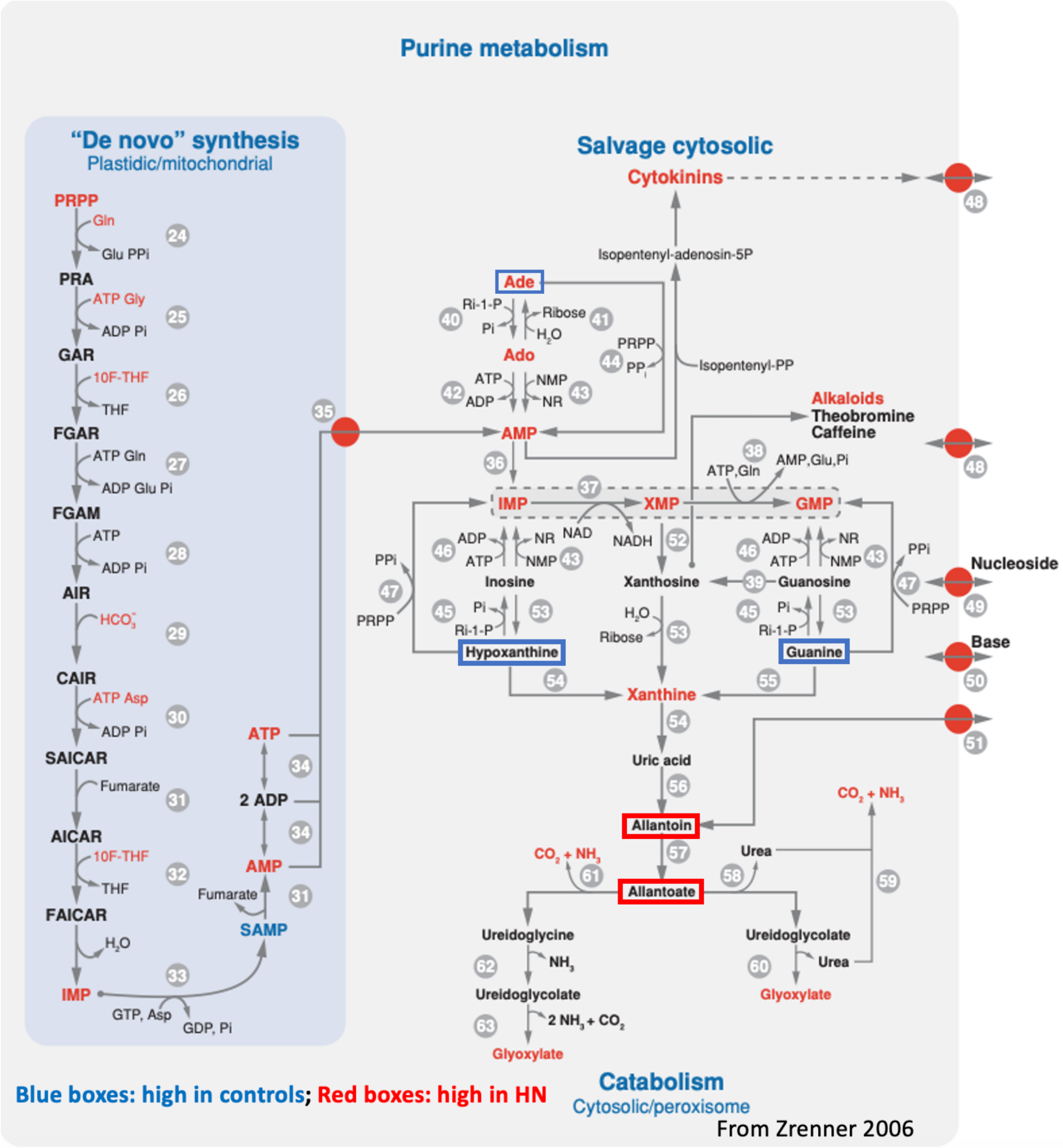

Fig. S8. Purine *de novo* synthesis and catabolism in plants

(from Zrenner *et al*. 2006)

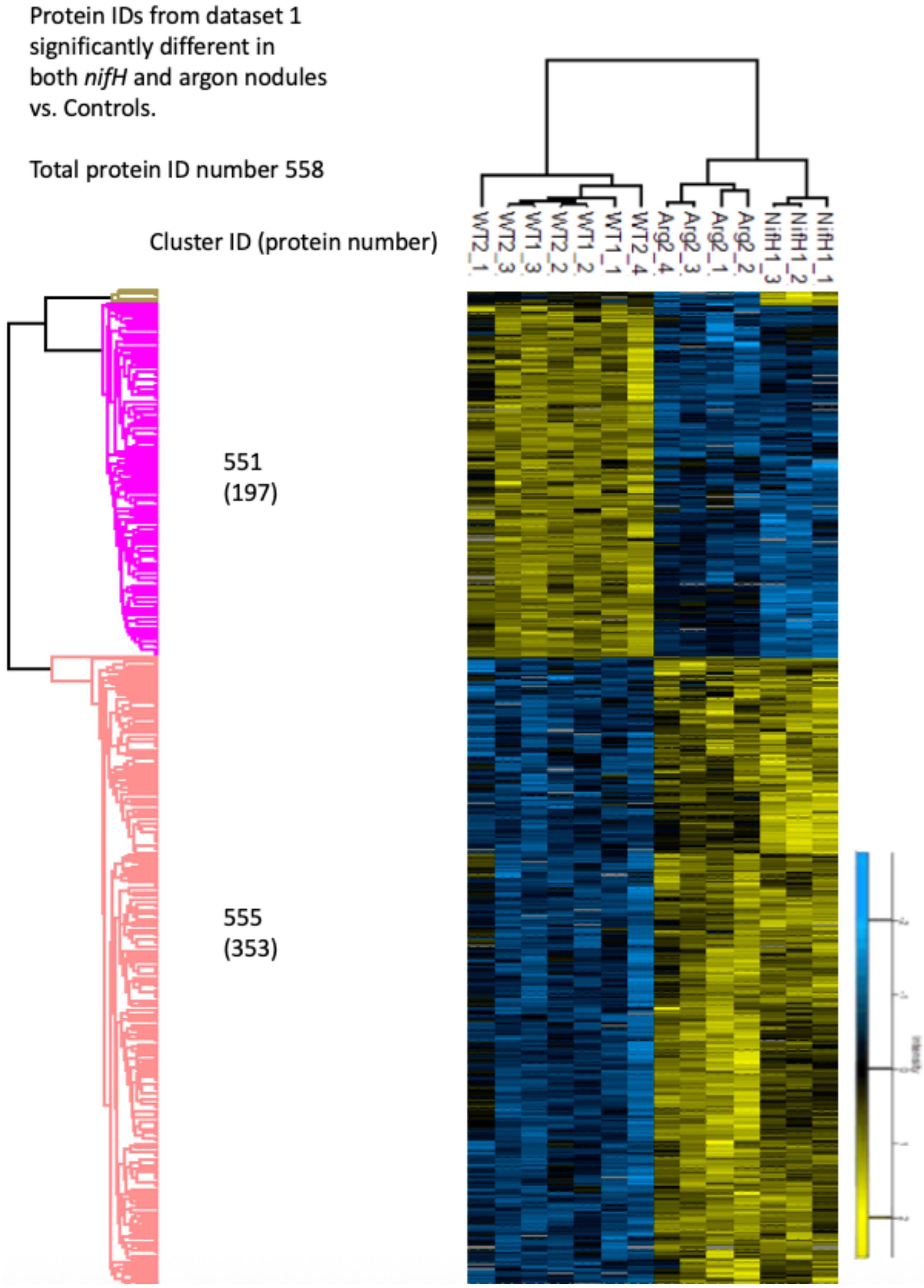

Fig. S9 Global clustering of proteomics results from label-free analysis of *M. truncatula* nodules during continuous treatments with *nifH* and argon (Dataset 1).

Proteins were clustered according to significant upregulation (cluster 555) or downregulation (cluster 551) in both, *nifH* and argon nodules, relative to controls (see **Table S1**).

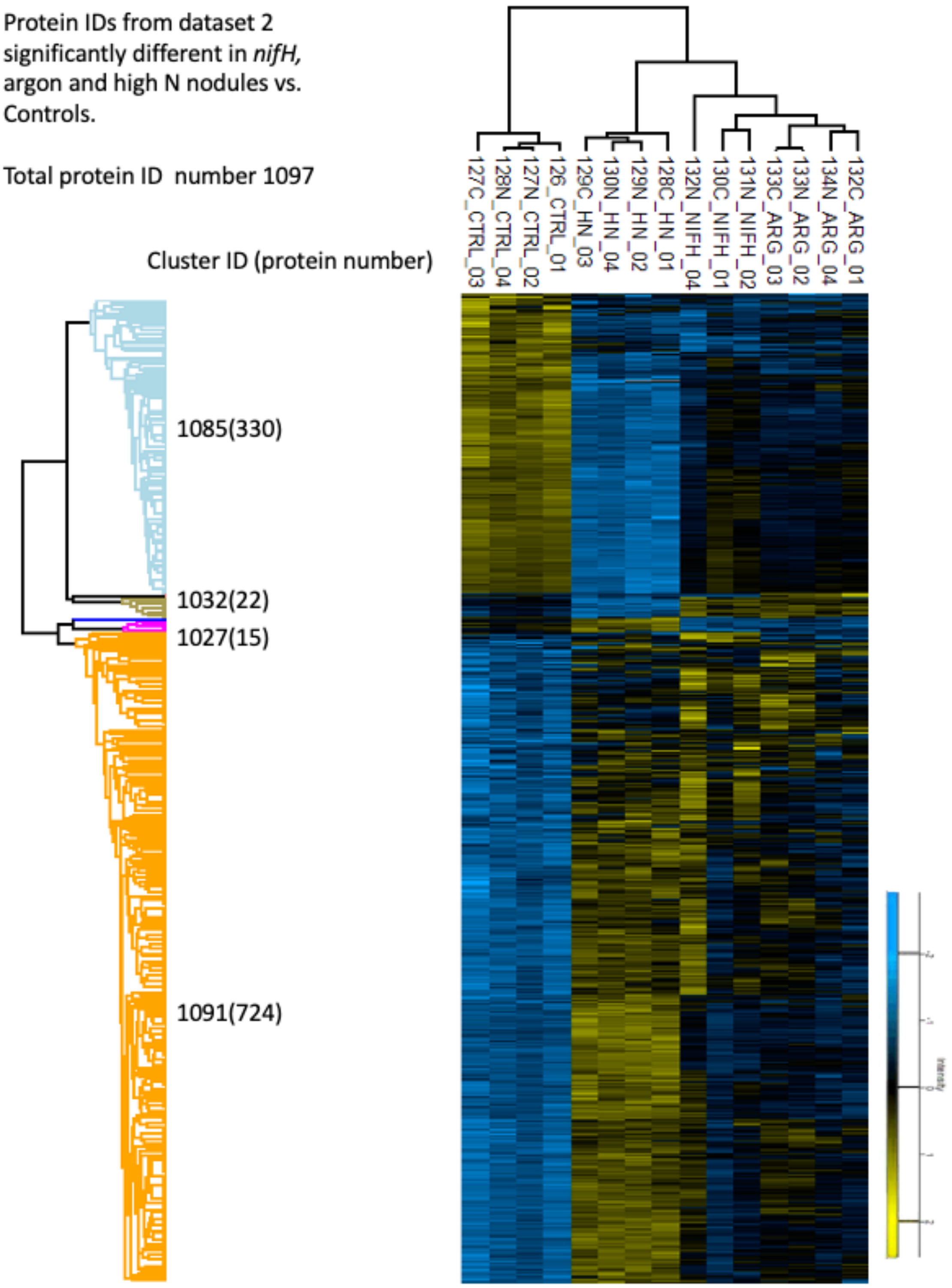

Fig. S10 Global clustering of proteomics results from TMT-labelled analysis of *M. truncatula* nodules from treatments with *nifH*, argon, or high N (Dataset 2).

Proteins were clustered according to significant upregulation (cluster 1091) or downregulation (cluster 1085) in all three sanctioning conditions (*nifH*, argon, high N) relative to controls (see **Table S2**).

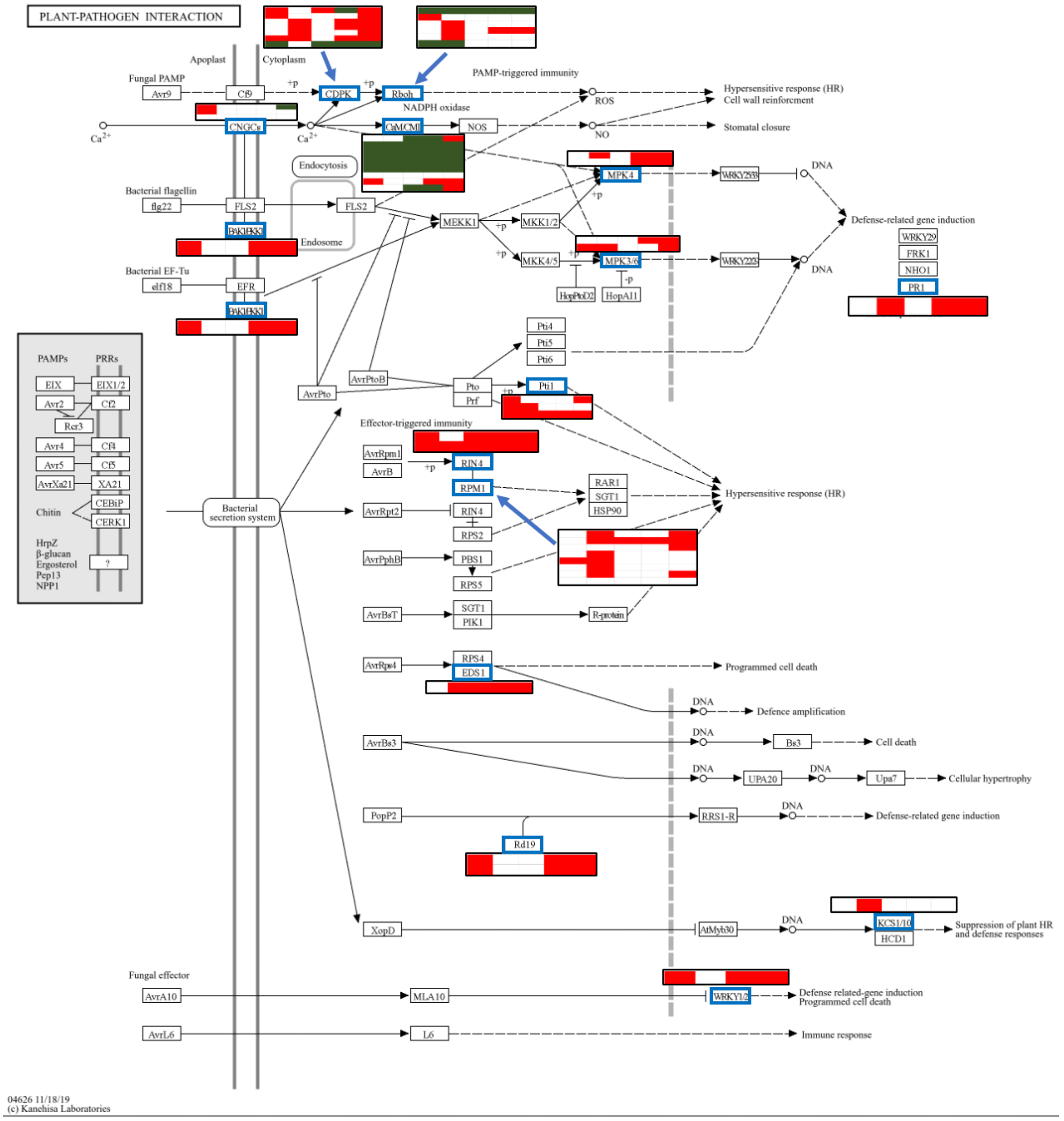

Dataset1		Dataset2		
log2 nifH-wt	log2 arg-cont.	log2 nifH-wt	log2 arg-cont.	log2 HN-wt

upregulated (red)	FDR<0.05
downregulated (green)	

Fig. S11 Mapping of *M. truncatula* defense-related proteins on the KEGG pathway “Plant-pathogen interaction”.

GO-terms that were significantly enriched are highlighted with blue boxes and associated with blocks representing the individual proteins in the respective GO-terms and their induction (red) or reduction (green) in all three sanctioning conditions relative to the controls.

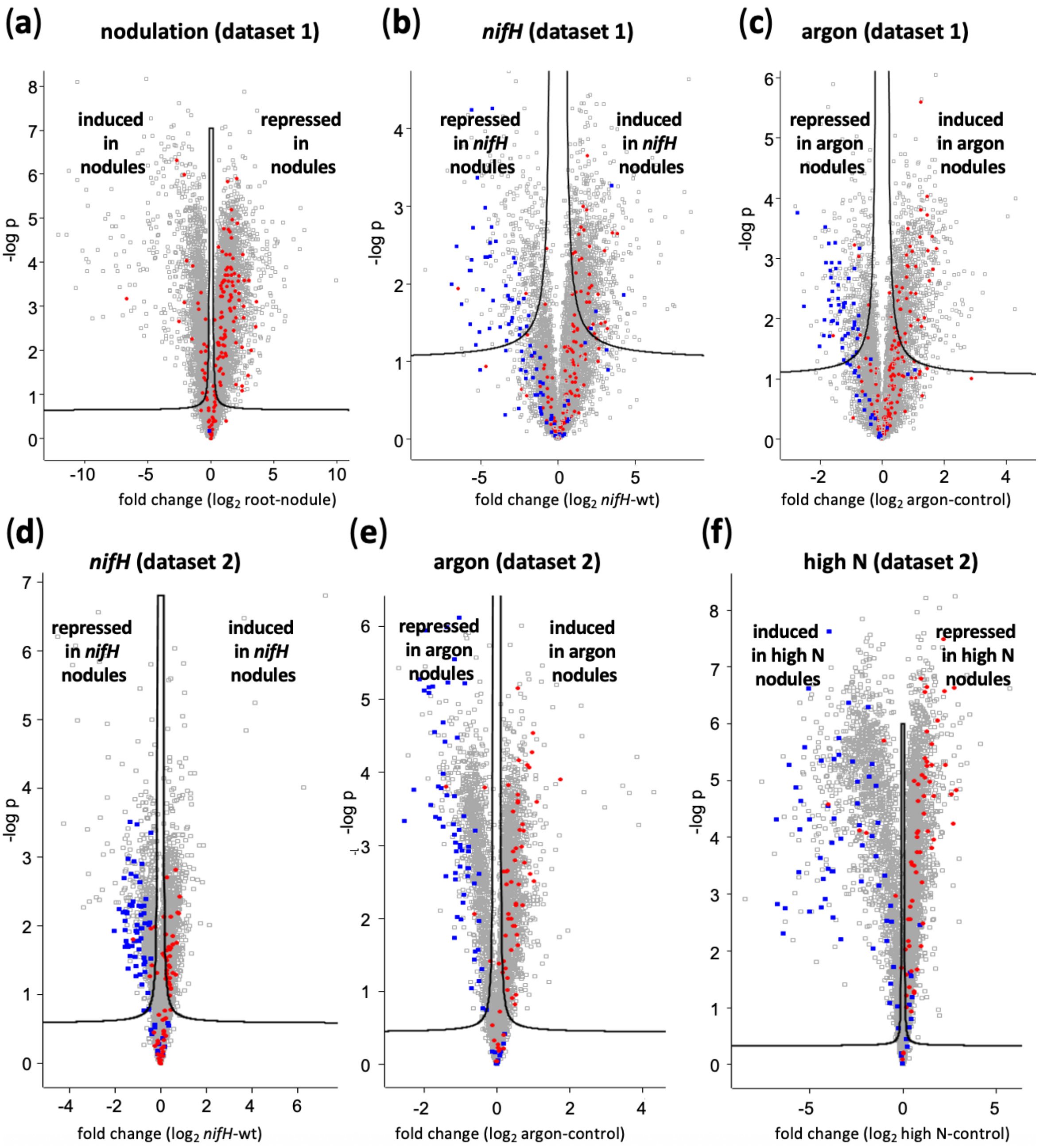

Fig. S12 Relative expression of NCRs and defense-related proteins in nodules of *M. trucatula*.

Volcano plot analysis of defense-related proteins (red dots) and NCR peptides (blue dots) according to the log2-fold change of non-fixing vs. controls (*x*-axis) and the -log10 *p*-value for the significance of the change (*y*-axis). Comparisons involved (**a**) *M. truncatula* roots *versus* nodules (Dataset 1), (**b**) *nifH* nodules *vs.* wt nodules (Dataset 1), (**c**) argon *vs.* control nodules (Dataset 1), and the three sanctioning treatments of Dataset 2, i.e. *nifH* nodules *vs.* wt nodules (**d**), argon *vs.* control nodules (**e**), and high N *vs.* control nodules (**f**), as indicated.

(a)

MAQRTHVPKFGNWESNDNVPYTAYFDKARKGRTGTKMINPNDPEENVDLVLDNSSS
DHPPPSNTKPRANSEDL**S**GKGLVRSTIE**S**HK**S**LDGDPKQYVD**S**PARHDNA**S**NR**S**SN
D**ST**PRLGVGSADNRRRPSRQSTAGSEH**S**VERSPLHRQARAPA**GRDSP****S**WEGKNNSY
D**SS**HG**T**PGRSRLRPANRGDE**T**PDKGAAVPKFGDWDVSNPASADGY**T**HIFNKVREER
QGGAGHAPG**T**PNERPHVIRNQNNDKAQCCCFAWGKK

(b)

Peptide sequence	Master Protein Accessions	Modifications in Master Proteins	Description	p-val. nifH vs wt	q-val. nifH vs wt	log2 nifH-wt	p-val. arg. vs cont.	q-val. arg. vs cont.	log2 arg-cont.	p-val. HN vs wt	q-val. HN vs wt	log2 HN-wt
STIESHK	A0A072TXB9	A0A072TXB9 1xPhospho [S85(100)]	RIN4	3.66	0.00	**3.49**	4.21	0.00	**4.67**	2.22	0.00	**1.91**
APAGRDSPSWEGK	A0A072TXB9	A0A072TXB9 1xPhospho [S158(93.5)]	RIN4	0.26	0.81	-0.20	1.67	0.03	**1.29**	2.09	0.01	**-1.43**
QSTAGSEHSVERSPLHR	A0A072TXB9	A0A072TXB9 1xPhospho [S140(98.3)]	RIN4	0.89	0.06	-1.48	0.01	0.98	-0.02	0.85	0.01	**-1.42**

Fig. S13 Phosphorylation pattern of *M. truncatula* RIN4 during sanctioning.

(**a**) Amino acid sequence of MtRIN4; All phosphorylation sites are highlighted in color. Induced and reduced phosphorylations are highlighted in red and blue, respectively. The conserved phosphosite in the legume-specific GRDSP motif (Toth *et al*. 2023) is highlighted in green. (**b**) Quantification of the phosphorylation levels of the three phosphosites highlighted in red, green, and blue, respectively, in (**a**). The relative difference in expression level (difference -log2) is highlighted in the respective colors as in (**a**).

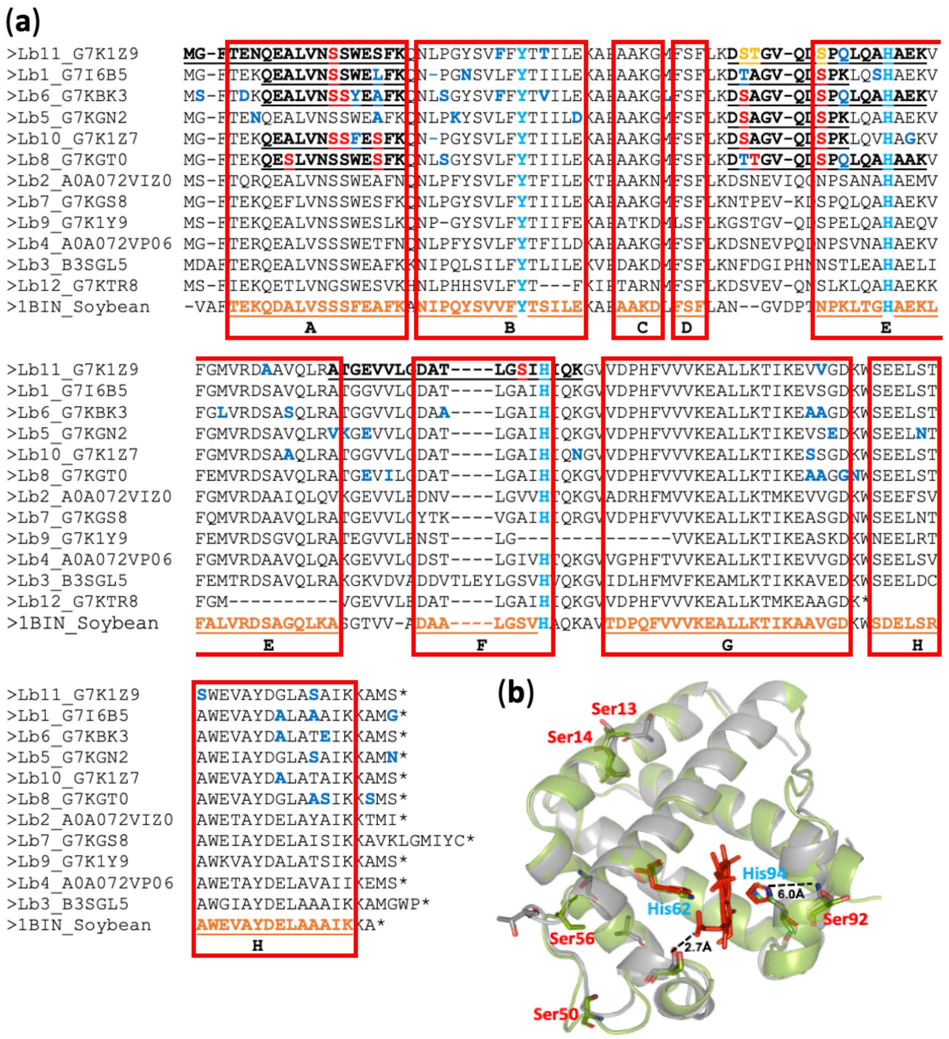

(c)

Gene IDs (old)	Gene IDs (new)	Protein ID	Larrainzar et al., (2020)	Jiang et al., (2021)
Medtr1g011540	1g0148751	P27993	Lb1	LgHb7
Medtr1g049330	1g0170851	A0A072VIZ0	Lb2	LgHb4
Medtr1g090810	1g0197501	B3SGL5	Lb3	LgHb11
Medtr1g090820	1g0197531	A0A072VP06	Lb4	LgHb10
Medtr5g041610	5g0417631	G7KGN2	Lb5	LgHb6
Medtr5g066070	5g0427351	G7KBK3	Lb6	LgHb9
Medtr5g080400	5g0435611	G7KGS8	Lb7	LgHb3
Medtr5g080440	5g0435621	G7KGT0	Lb8	LgHb1
Medtr5g080900	Not annotated	G7K1Y9	Lb9	LgHb5
Medtr5g081000	5g0435981	G7K1Z7	Lb10	LgHb8
Medtr5g081030	5g0435991	G7K1Z9	Lb11	LgHb2
Medtr7g110180	7g0270491	G7KTR8	Lb12	LgHb12

Fig. S14 Analysis of Lb phosphorylation patterns during sanctioning in *M. truncatula*.

(**a**) Phosphorylation patterns in the 12 aligned Lb proteins of *M. truncatula* (Lb nomenclature according to Larrainzar *et al*., (2020); see panel **c**). The six top Lb sequences (Lb11, Lb1, Lb6, Lb5, Lb10, and Lb8) are phosphorylated in a consistent pattern in helix A (Ser13, Ser14), between helices D and E (Ser50, Ser56), and in helix F (Ser92). Red residues indicate phosphosites; orange residues in Lb11 belong to a phosphorylated tryptic peptide with several Ser and Thr residues, in which case the exact phophosite is unknown, hence, all three potential phosphosites are highlighted in orange. Conserved His and Tyr residues involved in heme complexing (Kundu *et al*., 2003) are highlighted in light blue, non-conserved

residues among the six phosphorylated Lbs are highlighted in dark blue. Helical domains A to H (according to Raimondi *et al*., 2017) are indicated with red boxes.

(**b**) 3D-model of *M. truncatula* Lb11 (G7K1Z9; light green) superimposed on the soybean orthologue GmLba (pdb_1BIN; light grey) with the indicated P-sites at Ser13, Ser14, Ser50, Ser56, and Ser92 to allow to predict their position in the protein and their potential effects on protein function. Phosphorylation of Ser13 and Ser14 on the surface of helix A is probably not directly affecting Lb function. Phosphorylation of Ser50 and Ser56 may affect a hinge region between helices D and E which could potentially alter the environment of the heme group (red structure). Phosphorylated Ser92 is located next to a conserved coordinating His (blue His94 in (**a**)), indicating that it may directly alter iron coordination and therefore O_2 binding.

(**c**) Lb nomenclature used in this study according to Larrainzar *et al*., (2020), and in relation to Jiang *et al*. (2021).

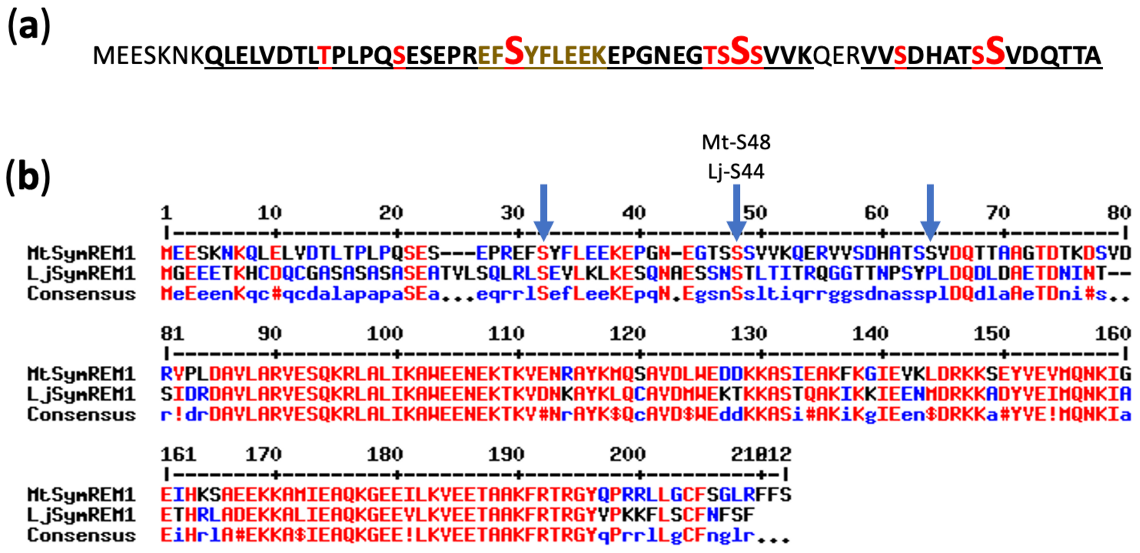

Fig. S15 Phosphorylation patterns of *M. truncatula* SymREM1 during sanctioning.

(**a**) Phosphosites in the N-terminal intrinsically disordered region of *M. truncatula* SymREM1 are highlighted in red. Significantly induced phosphorylation levels among the top 100 phosphopeptides (Table S5) are highlighted with increased font size. (**b**) Alignment of *M. truncatula* and *L. japonicus* SymREM1 amino acid sequence showing the N-terminal intrinsically disordered domain of approximately 80 amino acids, which shows a very low level of sequence conservation. Nevertheless, two of the three phosphorylated Ser residues are conserved (arrows), in particular Ser48 (Ser44 in *L. japonicus*), which is phosphorylated by NFR1 and SYMRK in *L. japonicus* (Toth *et al*., 2012). (d) Lb nomenclature used here based on Larrainzar et al., (2020), and differences relative to previous

Methods S1 Click here to enter text.

Metabolomics analysis

Chromatographic separation was performed on a Vanquish Flex UPLC system (Thermo Fisher Scientific) interfaced with a Q-Exactive Plus mass spectrometer (Thermo Fisher Scientific), using a heated electrospray ionization (HESI-II) source. Thermo Scientific Xcalibur 3.1 software was used for instrument control. The LC conditions were as follows: column, Waters Atlantis Premier BEH Z-HILIC, 100 × 2.1 mm, 1.7 µm; mobile phase, (A) water with 10 mM ammonium formate and 0.1% formic acid; (B) acetonitrile with 0.1% formic acid; flow rate, 600 µL/min; injection volume, 2 µL; gradient, linear gradient of 95%−60% B over 10 min and isocratic at 60% B for 2 min, reconditioning for 3 min. The optimized HESI-II parameters were as follows: source voltage, 3.5 kV (pos) / 2.5 kV (neg); sheath gas flow rate (N_2), 55 units (pos/neg); auxiliary gas flow rate, 15 units (pos/neg); spare gas flow rate, 3.0 (pos/neg); capillary temperature, 350°C (pos) / 275°C (neg), S-Lens RF Level, 50 (pos/neg); Probe heater temperature 450°C (pos/neg). The mass analyzer was calibrated using a mixture of caffeine, methionine–arginine–phenylalanine–alanine–acetate, sodium dodecyl sulfate, sodium taurocholate, and Ultramark 1621 in an acetonitrile/methanol/water solution containing 1% formic acid by direct injection. The data-dependent tandem mass spectrometry (MS/MS) events were performed on the three most intense ions detected in full scan MS (Top3 experiment). The MS/MS isolation window width was 1 Da, and the stepped normalized collision energy was set to 15, 30, and 45 units. In data-dependent MS/MS experiments, full scans were acquired at a resolution of 35,000 Full Width at Half Maximum (FWHM) (at *m*/*z* 200) and MS/MS scans at 17,500 FWHM both with an automatically determined maximum injection time. After being acquired in an MS/MS scan, parent ions were placed in a dynamic exclusion list for 3.0 s.

Data pre-treatment

The MS data were converted from .RAW (Thermo) standard data format to .mzML format using the MSConvert software, part of the ProteoWizard package (Chambers *et al.*, 2012). The converted files were treated using the MZmine v 4.1 (Pluskal *et al.*, 2010). The parameters

were adjusted as follows: the centroid mass detector was used for mass detection with the noise level set to 2.0E4 for the MS level set to 1, and to 0 for the MS level set to 2. The ADAP chromatogram builder was used and set to a minimum group size of scans of 5, minimum group intensity threshold of 2.0E4, minimum highest intensity of 2.0E4, and m/z tolerance of 12 ppm (Myers *et al.*, 2017). For chromatogram deconvolution, the algorithm used was the wavelets (ADAP). The intensity window signal-to-noise (S/N) was used as an S/N estimator with a S/N ratio set at 15, a minimum feature height at 2.0E4, a coefficient area threshold at 80, a peak duration range from 0.02 to 1.00 min and the retention time (RT) wavelet range from 0.02 to 0.05 min. Corresponding MS2 was paired with the following parameters (0.025 Da and 0.15 min). Isotopes were detected using the isotope peaks grouper with a *m/z* tolerance of 8 ppm, an RT tolerance of 0.08 min (absolute), the maximum charge set at 4, and the lowest *m/z* was used as the representative isotope. Peak alignment was performed using the join aligner method (*m/z* tolerance at 12 ppm), absolute RT tolerance 0.08 min, weight for *m/z* at 30, and weight for RT at 30. The aligned feature list (1007 and 1154 features, respectively for positive and negative ionization modes) were exported using the export to Global Natural Product Social Molecular Networking (GNPS) Feature-Based Molecular Network (FBMN) module.

Molecular networking

To analyze the spectral diversity of the profile collection, a molecular network (MN) was created on the GNPS website (http://gnps.ucsd.edu) using the. mgf spectra file generated at the previous step (Wang *et al.*, 2016). The precursor ion mass tolerance was set to 0.02 Da and an MS/MS fragment ion tolerance of 0.02 Da. A network was then created where edges were filtered to have a cosine score above 0.7 and more than six matched peaks. Further, edges between two nodes were kept in the network if and only if each of the nodes appeared in each other's respective top 10 most similar nodes. Finally, the maximum size of a spectral family was set to 100, and the lowest-scoring edges were removed from molecular families until the molecular family size was below this threshold. The spectra in the network were then searched against GNPS spectral libraries. All matches kept between network spectra and library spectra were required to have a score above 0.7 and at least six matched peaks. The resulting MN jobs are available online at https://gnps.ucsd.edu/ProteoSAFe/status.jsp?task=74a7bc6828294e8c95bdba312f1b58b7

(positive ionization mode) and https://gnps2.org/status?task=9be4451a46b74244bf07da72cba2965b (negative ionization mode). The converted mass spectrometry data files, the corresponding metadata table, and the metabolite annotation results table, together with a Cytoscape file corresponding to the full MN annotated with the experimental and theoretical spectral matches are available under the MassIVE ID https://doi.org/doi:10.25345/C57P8TR88.

Metabolite annotation / Experimental spectral libraries search

The full spectral dataset corresponding to the extracts collection has been uploaded on the MassIVE repository https://doi.org/doi:10.25345/C57P8TR88, and a metabolite annotation workflow were automatically carried against the Global Natural Products Social Molecular Networking (GNPS) experimental spectral libraries: https://gnps.ucsd.edu/ProteoSAFe/result.jsp?task=74a7bc6828294e8c95bdba312f1b58b7&view=group_by_compound (positive ionisation mode) and https://gnps2.org/result?task=a10155676345444dba86da0964c1f43f&viewname=librarymatches&resultdisplay_type=task (negative ionization mode).

Theoretical spectral library search

In addition to experimental spectral libraries search we have shown that spectral matching against theoretical spectral libraries of natural products was an efficient way to cover a much wider, yet relevant, spectral space (Allard *et al.*, 2016). Furthermore, we showed that taking into account the taxonomical distance between the biological source of the candidate structure and the biological source of the annotated extracts greatly improved the overall quality of the annotation results (Rutz *et al.*, 2019) . Thus, in addition to the spectral search performed at the molecular networking step against publicly available spectral libraries (see previous section) a taxonomically informed metabolite annotation was performed. For this, we first established a large theoretical spectral database of natural products following a previously established metabolite annotation workflow. This spectral database and associated biological sources metadata were constructed using chemical structure and information compiled during the LOTUS Initiative's first project aiming to establish an open and evolutive resource compiling natural products and biological occurrences (Rutz *et al.*, 2022a; Rutz *et al.*, 2022b). The theoretical spectral database is publicly available (Allard *et al.*,

2022). The biological sources metadata are available online (Rutz *et al.*, 2022a; Rutz *et al.*, 2022b). The taxonomically informed metabolite annotation was performed using the met_annot_enhancer scripts version v0.1: https://github.com/mandelbrot-project/met_annot_enhancer/releases/tag/v0.1. The parameters used for the taxonomically informed metabolite annotation process and the resulting tables can be found at https://doi.org/doi:10.25345/C57P8TR88. Sirius (Dührkop *et al.*, 2019) (v.5.5.7) and CANOPUS (Feunang *et al.*, 2016; Dührkop *et al.*, 2021; Kim *et al.*, 2021) were also employed to proceed to metabolite annotation and attribution of chemical classes to MSMS spectra.

Statistical analysis

For metabolomics analysis, scripts to proceed to multivariate unsupervised and supervised analysis and highlight the discriminant features among the different conditions are available at the following GitHub repository https://github.com/mapp-metabolomics-unit/biostat_toolbox. They were built upon the structToolbox R package (Lloyd *et al.*, 2020). For proteomics, statistical analysis was performed in Perseus by pairwise comparison using t-test, the FDR<0.05 was controlled by permutation-based method. Boxplots in figures represent the median and the interquartile range; whiskers indicate the data range excluding outliers. Significance of differences was tested with one-way ANOVA followed by Tukey's post-hoc test.

Proteome sample preparation for label-free quantification (Dataset 1)

Protein extraction was performed according to Marx *et al.* (2016). Briefly, 100 mg of plant material was ground in liquid nitrogen to a fine powder. One ml of extraction buffer, 290 mM sucrose, 250 mM Tris-Cl, pH 7.6, protease inhibitor cocktail was added, well mixed, and sonicated. After removing of cell debris by centrifugation, one volume of chloroform was added and samples were mixed thoroughly by vortexing. After adding 3 volumes of water and mixing, samples were centrifuged 5 min at 14,000*g* at 4°C. The upper phase was removed and 3 volumes of methanol was added to the lower phase and interphase and mixed well. After centrifugation, the pellet was washed 3 times with 80% acetone and dried at RT. Pellets were resuspended in 8 M urea in 50 mM Tris-Cl, pH 8, reduced with DTT (final concentration 1 mM, 30 min at RT) and alkylated with IAA (final concentration 5.5 mM, 30 min at RT in the dark).

Proteome sample preparation for tandem mass tag-based quantification (Dataset 2)

After extraction of metabolites (see above), samples were lyophilized before resuspending pellet in 6 M guanidine hydrochloride (GdnHCl) in 100 mM HEPES, pH 8. After mixing, samples were sonicated and cell debris were removed by centrifugation. Proteins were reduced with TCEP (final concentration 10 mM, 1 h at 55°C) and alkylated with IAA (final concentration 22 mM, 30 min at RT). After acetone precipitation, proteins were resuspended in small volume of 2.5 M GdnHCl in 100 mM HEPES, pH 8 and 150 µg protein of each sample were digested overnight with LysC and trypsin (ratio 50:1, final volume of 100 µl, final GdnHCl concentration of 0.75 M). TMTpro labeling was performed according to the manufacturer's recommendations. After combining same amount of each TMT channel, peptide mixture was fractionated by HpH reverse phase chromatography (see above) resulting in eight fractions. Phosphopeptide enrichment was performed according to (Post *et al.*, 2017) using Fe(III) cartridges on Bravo liquide handling system (Agilent).

LC-MS/MS measurements were performed on an Exploris 480 mass spectrometer coupled to an EasyLC 1200 nanoflow-HPLC (all Thermo Scientific). Peptides were seperated on a fused silica HPLC-column tip (I.D. 75 µm, New Objective, self-packed with ReproSil-Pur 120 C18-AQ, 1.9 µm (Dr. Maisch; https://dr-maisch.com) to a length of 20 cm) using a gradient of A (0.1% formic acid in water) and B (0.1% formic acid in 80% acetonitrile in water): samples were loaded with 0% B with a flow rate of 600 nl/min; peptides were separated by 7%–38% B within 122 min with a flow rate of 250 nl/min. Spray voltage was set to 2.3 kV and the ion-transfer tube temperature to 250°C; no sheath and auxiliary gas were used. Mass spectrometers were operated in the data-dependent mode; after each MS scan (m/z = 370–1750; resolution: 120,000) a maximum of twenty MS/MS scans were performed using an isolation window of 0.7, a normalized collision energy of 32%, a target AGC of 50% and a resolution of 45,000. MS raw files were analyzed using ProteomeDiscoverer (version 2.5, Thermo Scientific) using a Uniprot full-length *Medicago truncatula* database and a *Sinorhizobium meliloti* database. Carbamidomethylcysteine was set as fixed modification and protein amino-terminal acetylation and oxidation of methionine were set as variable modifications. The MS/MS tolerance was set to 0.6 Da and three missed cleavages were allowed using trypsin/P as enzyme specificity. TMTpro correction factors were used. Exported normalized abundances for each channel were further analyzed using Perseus software (Tyanova *et al.*, 2016).

Proteomic analysis

After protein extraction, equal amounts of proteins per sample were digested with LysC (ratio 50:1, for 3 h, at RT, final urea concentration 4 M) and trypsin (ratio 50:1, overnight, at RT, final urea concentration <1 M). Next day, the samples were acidified using 50% TFA (final concentration app. 0.5%, pH<2) and centrifuged at 1500g for 10 min to remove precipitations. Peptides were purified by SPE using HR-X columns in combination with C18 cartridges (Macherey- Nagel; https://www.mn-net.com): wash buffer, 0.1% formic acid in deionized water; elution buffer, 80% acetonitrile, and 0.1% formic acid in deionized water. Elutes were frozen in liquid nitrogen and lyophilized overnight. Purified peptides were fractionated by HpH reversed phase chromatography (Batth *et al.*, 2014). Briefly, dry peptide powder was suspended in 400 µl 5% ammonium hydroxide and fractionated using a Waters XBridge BEH130 C18 column (3.5 µm 4.6 × 250 mm) on a Ultimate 3000 HPLC (Thermo Scientific). The flow rate of the mobile phase was 1 ml/min. HpH buffer A contained 10 mM ammonium formate in deionized water and buffer B contained 10 mM ammonium formate and 90% acetonitrile deionized water. Both buffers were adjusted to pH 10 with ammonium hydroxide. Peptide fractions were acidified, frozen in liquid nitrogen, and lyophilized overnight.

LC-MS/MS measurements were performed on an QExactive HFX mass spectrometer coupled to an EasyLC 1000 nanoflow-HPLC (all Thermo Scientific). Peptides were separated on a fused silica HPLC-column tip (I.D. 75 µm, New Objective, self-packed with ReproSil-Pur 120 C18-AQ, 1.9 µm (Dr. Maisch; https://dr-maisch.com) to a length of 20 cm) using a gradient of A (0.1% formic acid in water) and B (0.1% formic acid in 80% acetonitrile in water): samples were loaded with 0% B with a flow rate of 600 nl/min; peptides were separated by 5%–30% B within 85 min with a flow rate of 250 nl/min. Spray voltage was set to 2.3 kV and the ion-transfer tube temperature to 250°C; no sheath and auxiliary gas were used. Mass spectrometers were operated in the data-dependent mode; after each MS scan (m/z = 370-1750; resolution: 120,000) a maximum of twelve MS/MS scans were performed using an isolation window of 1.6, a normalized collision energy of 28%, a target AGC of 1e5 and a resolution of 30,000. MS raw files were analyzed using MaxQuant software using a Uniprot full-length *M. truncatula* database and a *S. meliloti* database. Carbamidomethylcysteine was set as fixed modification and protein amino-terminal acetylation and oxidation of methionine were set as variable modifications. The MS/MS tolerance was set to 20 ppm and three missed cleavages were

allowed using trypsin/P as enzyme specificity. Peptide and protein FDR based on a forward-reverse database were set to 0.01, minimum peptide length was set to 7, and minimum number of peptides for identification of proteins was set to one, which must be unique. The "match-between-run" option was used with a time window of 0.7 min. MaxQuant results were analyzed using Perseus. The mass spectrometry proteomics data have been deposited to the ProteomeXchange Consortium via the PRIDE partner repository with the dataset identifiers PXD065143, PXD065225, and PXD065245.
LLC).

Staining procedures and microscopy

Life-and-dead staining was performed as described (Nicoud *et al.*, 2021): Briefly, fresh nodules were embedded in 6% agar and 0.2M sucrose. Nodule sections were obtained using the vibratome HM650S. at a thickness of approximately 70 mm. The nodule sections were stored in 0.2M sucrose until staining. The staining solution was prepared in 0.2M sucrose with 5 mM Syto9 and 10 mM Propidium Iodide (PI). Sections were stained for 20–30 minutes and directly analyzed using a Leica SPE-II laser scanning confocal microscope. Syto9 signal was excited using the 488 nm laser line, and PI was excited with the 550 nm laser line.
Basic fuchsin staining: Nodules were collected and fixed in 4% paraformaldehyde (PFA) in 1x PBS overnight. Fixed nodules were kept in PFA until processing and then washed three times with 1x PBS. The nodules were embedded in 6% agar and sectioned using the vibratome HM650S. Sections were stained with 0.2% basic fuchsin in ClearSee (Kurihara *et al.*, 2015) for 2 days. Before analysis, the sections were washed in ClearSee and mounted on slides. Basic fuchsin was imaged on a Leica SPE-II laser scanning confocal microscope using the 561 nm laser line for excitation, and detection between 600–650 nm.
Starch staining was performed by immersing intact nodules in Lugol staining solution (Sigma) for 4 minutes, followed by longitudinal hand-sectioning and microscopy in a Leica MZFLIII stereoscope equipped with a Nikon camera (Digital Sight DS-U1).

Transmission electron microscopy (TEM)

Nodule samples were fixed by high-pressure freezing and cryo-substitution. Briefly, nodules were placed in an aluminum planchet of 3 mm in diameter with a cavity of 0.2 mm (Art.610, Wohlwend GmbH, Sennwald, Switzerland) filled with Hexadecene and covered with a tap

planchet (Art.611, Wohlwend GmbH, Sennwald, Switzerland) and directly high-pressure frozen using the High-Pressure Freezing Instrument HPF Compact 02 (Wohlwend GmbH, Sennwald, Switzerland). Cryo-substitution and chemical fixation was carried out as follows: Dehydration and fixation was performed in a solution containing a mixture of osmium tetroxide 1.2% (EMS, Hatfield, PA) with glutaraldehyde 3% (EMS, Hatfield, PA) with uranyl acetate 0.5% (Sigma, St. Louis, MO) in acetone (Sigma, St Louis, MO, US) at graded temperatures (−90 °C for 60 h; from −90 °C to −60 °C in 30min; −60 °C for 24 h; from −60 °C to −30 °C in 30min; −30 °C for 24 h; from −30 °C to 0° in 3 h). This was followed by several washes in acetone and infiltration in Spurr resin (EMS, Hatfield, PA, US) at graded concentrations and temperatures (30% for 2h at 0 °C; 66% for 2h at 0 °C; 100% for 12 h at 20 °C) and finally polymerized for 48 h at 60 °C. For transmission electron microscopic (TEM) analysis, ultrathin sections (70 nm) were prepared on a Reichert-Jung Ultracut E. Contrasting was performed with 2% (w/v) uranyl acetate ($UO_2(CH_3COO)_2$) and lead citrate solution prepared according to (Reynolds, 1963). Images were acquired on a Philips Biotwin CM100 equipped with a digital camera (Morada Soft Imaging System).

Supplemental References

Allard P-M, Bisson J, Rutz A. 2022. ISDB: In silico spectral databases of natural products. *Zenodo* (https://zenodo.org/records/5607264).

Allard PM, Péresse T, Bisson J, Gindro K, Marcourt L, Pham VC, Roussi F, Litaudon M, Wolfender JL. 2016. Integration of molecular networking and *in-silico* MS/MS fragmentation for natural products dereplication. *Analytical Chemistry* **88**: 3317-3323.

Batth TS, Francavilla C, Olsen JV. 2014. Off-line high-pH reversed-phase fractionation for in-depth phosphoproteomics. *Journal of Proteome Research* **13**: 6176-6186.

Chambers MC, Maclean B, Burke R, Amodei D, Ruderman DL, Neumann S, Gatto L, Fischer B, Pratt B, Egertson J, *et al.* 2012. A cross-platform toolkit for mass spectrometry and proteomics. *Nature Biotechnology* **30**: 918-920.

Dührkop K, Fleischauer M, Ludwig M, Aksenov AA, Melnik AV, Meusel M, Dorrestein PC, Rousu J, Bocker S. 2019. SIRIUS 4: a rapid tool for turning tandem mass spectra into metabolite structure information. *Nature Methods* **16**: 299-302.

Dührkop K, Nothias LF, Fleischauer M, Reher R, Ludwig M, Hoffmann MA, Petras D,

Gerwick WH, Rousu J, Dorrestein PC, *et al.* 2021. Systematic classification of unknown metabolites using high-resolution fragmentation mass spectra. *Nature Biotechnology* **39**: 462-471.

Feunang YD, Eisner R, Knox C, Chepelev L, Hastings J, Owen G, Fahy E, Steinbeck C, Subramanian S, Bolton E, *et al.* 2016. ClassyFire: automated chemical classification with a comprehensive, computable taxonomy. *Journal of Cheminformatics* **8**: 61.

Jiang SY, Jardinaud MF, Gao JP, Pecrix Y, Wen JQ, Mysore K, Xu P, Sanchez-Canizares C, Ruan YT, Li QJ, et al. 2021. NIN-like protein transcription factors regulate leghemoglobin genes in legume nodules. *Science* **374**: 625-628.

Kim HW, Wang MX, Leber CA, Nothias LF, Reher R, Kang KB, van der Hooft JJJ, Dorrestein PC, Gerwick WH, Cottrell GW. 2021. NPClassifier: A deep neural network-based structural classification tool for natural products. *Journal of Natural Products* **84**: 2795-2807.

Kundu S, Trent JT, Hargrove MS. 2003. Plants, humans and hemoglobins. *Trends in Plant Science* **8**: 387-393.

Kurihara D, Mizuta Y, Sato Y, Higashiyama T. 2015. ClearSee: a rapid optical clearing reagent for whole-plant fluorescence imaging. *Development* **142**: 4168-4179.

Larrainzar E, Villar I, Rubio MC, Pérez-Rontomé C, Huertas R, Sato S, Mun JH, Becana M. 2020. Hemoglobins in the legume-*Rhizobium* symbiosis. *New Phytologist* **228**: 472-484.

Lloyd GR, Jankevics A, Weber RJM. 2020. Struct: an R/Bioconductor-based framework for standardized metabolomics data analysis and beyond. *Bioinformatics* **36**: 5551-5552.

Myers OD, Sumner SJ, Li SZ, Barnes S, Du XX. 2017. One step forward for reducing false positive and false negative compound identifications from mass spectrometry metabolomics data: new algorithms for constructing extracted ion chromatograms and detecting chromatographic peaks. *Analytical Chemistry* **89**: 8696-8703.

Nicoud Q, Barrière Q, Busset N, Dendene S, Travin D, Bourge M, Le Bars R, Boulogne C, Lecroë M, Jenei S, et al. 2021. *Sinorhizobium meliloti* functions required for resistance to antimicrobial NCR peptides and bacteroid differentiation. *Mbio* **12**: e00895-21.

Perez-Riverol Y, Bandla C, Kundu DJ, Kamatchinathan S, Bai JW, Hewapathirana S, John NS, Prakash A, Walzer M, Wang SB, *et al.* 2024. The PRIDE database at 20 years: 2025 update. *Nucleic Acids Research* **53**: D543-D553.

Pluskal T, Castillo S, Villar-Briones A, Oresic M. 2010. MZmine 2: Modular framework for

processing, visualizing, and analyzing mass spectrometry-based molecular profile data. *BMC Bioinformatics* **11**: 395.

Post H, Penning R, Fitzpatrick MA, Garrigues LB, Wu W, MacGillavry HD, Hoogenraad CC, Heck AJR, Altelaar AFM. 2017. Robust, sensitive, and automated phosphopeptide enrichment optimized for low sample amounts applied to primary hippocampal neurons. *Journal of Proteome Research* **16**: 728-737.

Raimondi D, Orlando G, Pancsa R, Khan T, Vranken WF. 2017. Exploring the sequence-based prediction of folding initiation sites in proteins. *Scientific Reports* **7**: 8826.

Reynolds ES. 1963. The use of lead citrate at high pH as an electron-opaque stain in electron microscopy. *Journal of Cell Biology* **17**: 208-212.

Rutz A, Bisson J, Allard P-M. 2022a. The LOTUS Initiative for Open Natural Products Research: frozen dataset union wikidata (with metadata). *Zenodo* (https://zenodo.org/records/7534071)

Rutz A, Dounoue-Kubo M, Ollivier S, Bisson J, Bagheri M, Saesong T, Ebrahimi SN, Ingkaninan K, Wolfender JL, Allard PM. 2019. Taxonomically informed scoring enhances confidence in natural products annotation. *Frontiers in Plant Science* **10**: 1329.

Rutz A, Sorokina M, Galgonek J, Mietchen D, Willighagen E, Gaudry A, Graham JG, Stephan R, Page R, Vondrásek J, *et al.* 2022b. The LOTUS initiative for open knowledge management in natural products research. *Elife* **11**: e70780.

Tyanova S, Temu T, Sinitcyn P, Carlson A, Hein MY, Geiger T, Mann M, Cox J. 2016. The Perseus computational platform for comprehensive analysis of (prote)omics data. *Nature Methods* **13**: 731-740.

Vasse J, Debilly F, Camut S, Truchet G. 1990. Correlation between ultrastructural differentiation of bacteroids and nitrogen-fixation in alfalfa nodules. *Journal Of Bacteriology* **172**: 4295-4306.

Wang MX, Carver JJ, Phelan VV, Sanchez LM, Garg N, Peng Y, Nguyen DD, Watrous J, Kapono CA, Luzzatto-Knaan T, *et al.* 2016. Sharing and community curation of mass spectrometry data with global natural products social molecular networking. *Nature Biotechnology* **34**: 828-837.
